# Supplementary material for: A new kentriodontid (Cetacea: Odontoceti) from the early to middle Miocene of the western North Pacific and a revision of kentriodontid phylogeny
Source: PeerJ. 2021 Feb 24;9:e10945. doi: 10.7717/peerj.10945 (PMC7912617; doi:10.7717/peerj.10945)
Supplement: Supplemental Information 1 — APPENDIX S1: Molecular tree and consensus trees resulting from our analysis APPENDIX S2: Institutional abbreviations and list of taxa and specimens examined APPENDIX S3: List of characters states used in phylogenetic analysis [file peerj-09-10945-s001.docx]

**Supplemental File 1**

**A new kentriodontid (Cetacea: Odontoceti) from the early to middle Miocene of the western North Pacific and a revision of kentriodontid phylogeny**

Zixuan Guo^*,1^ and Naoki Kohno^1,2^

^1^Faculty of Life and Environmental Sciences, University of Tsukuba, Tsukuba, Japan;

^2^Department of Geology and Paleontology, National Museum of Nature and Science, Tsukuba, Japan

APPENDIX S1: Molecular tree and consensus trees resulting from our analysis

APPENDIX S2: Institutional abbreviations and list of taxa and specimens examined

APPENDIX S3: List of characters states used in phylogenetic analysis

References


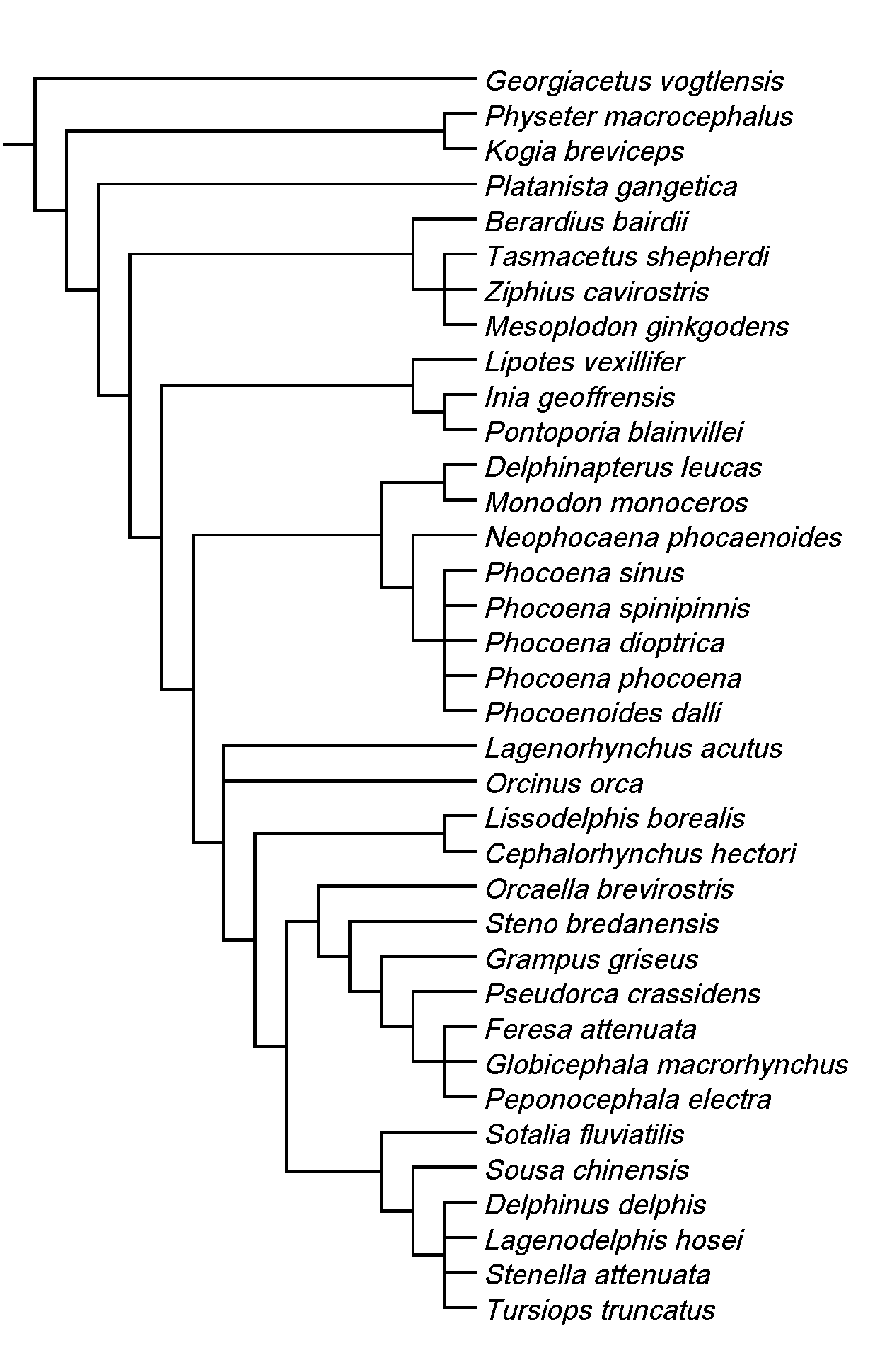
APPENDIX S1 (Figure S1). The topology of molecular phylogenetics of the extant cetaceans used as a backbone constraint. Modified from McGowen et al. (2009, 2011, 2020).


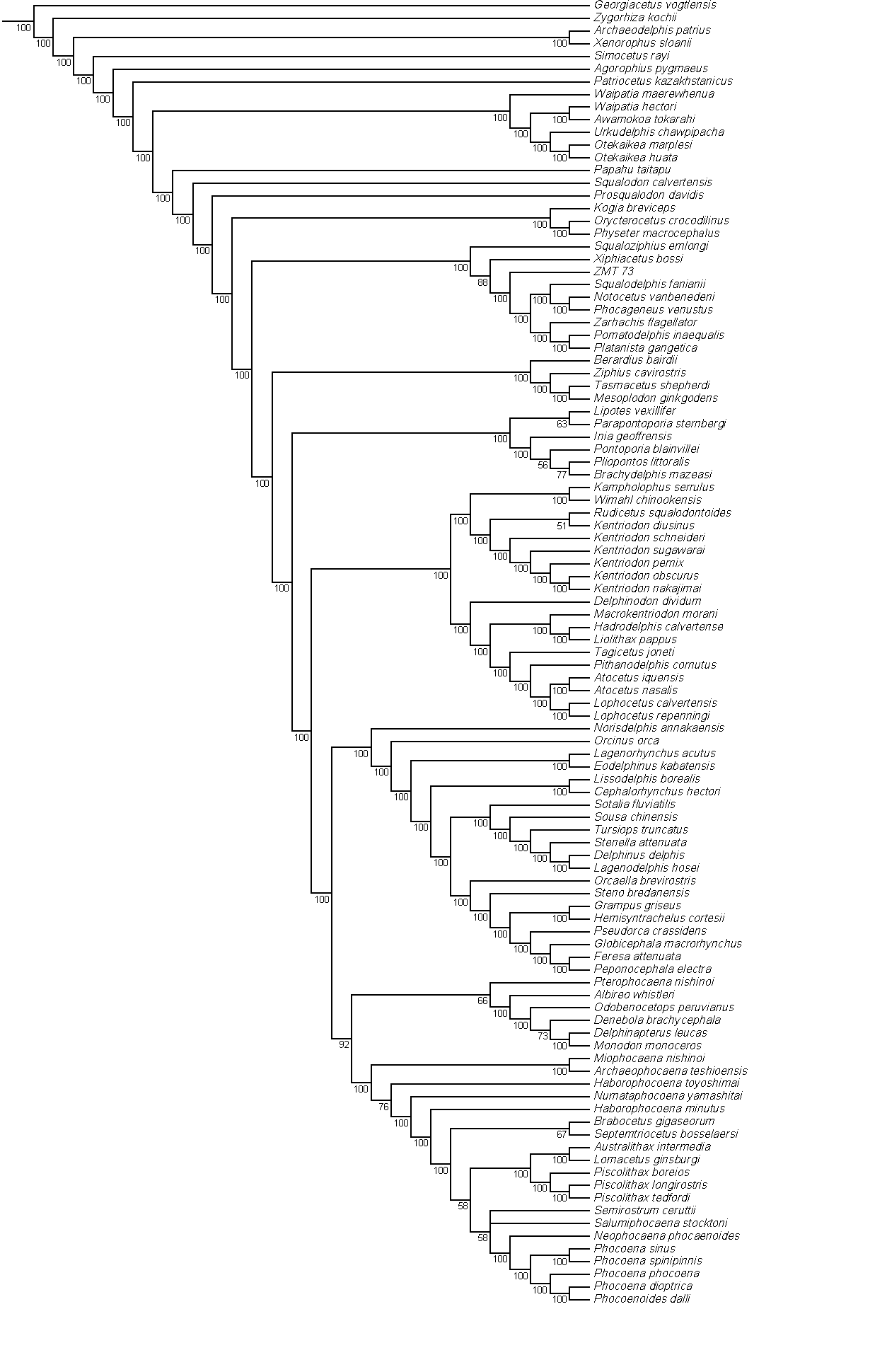
APPENDIX S1 (Figure S2). The 50% majority consensus tree resulting from 256 most parsimonious trees with tree constraint by the molecular tree. Values below the branches indicated probabilities of occurrence in the 256 most parsimonious trees.


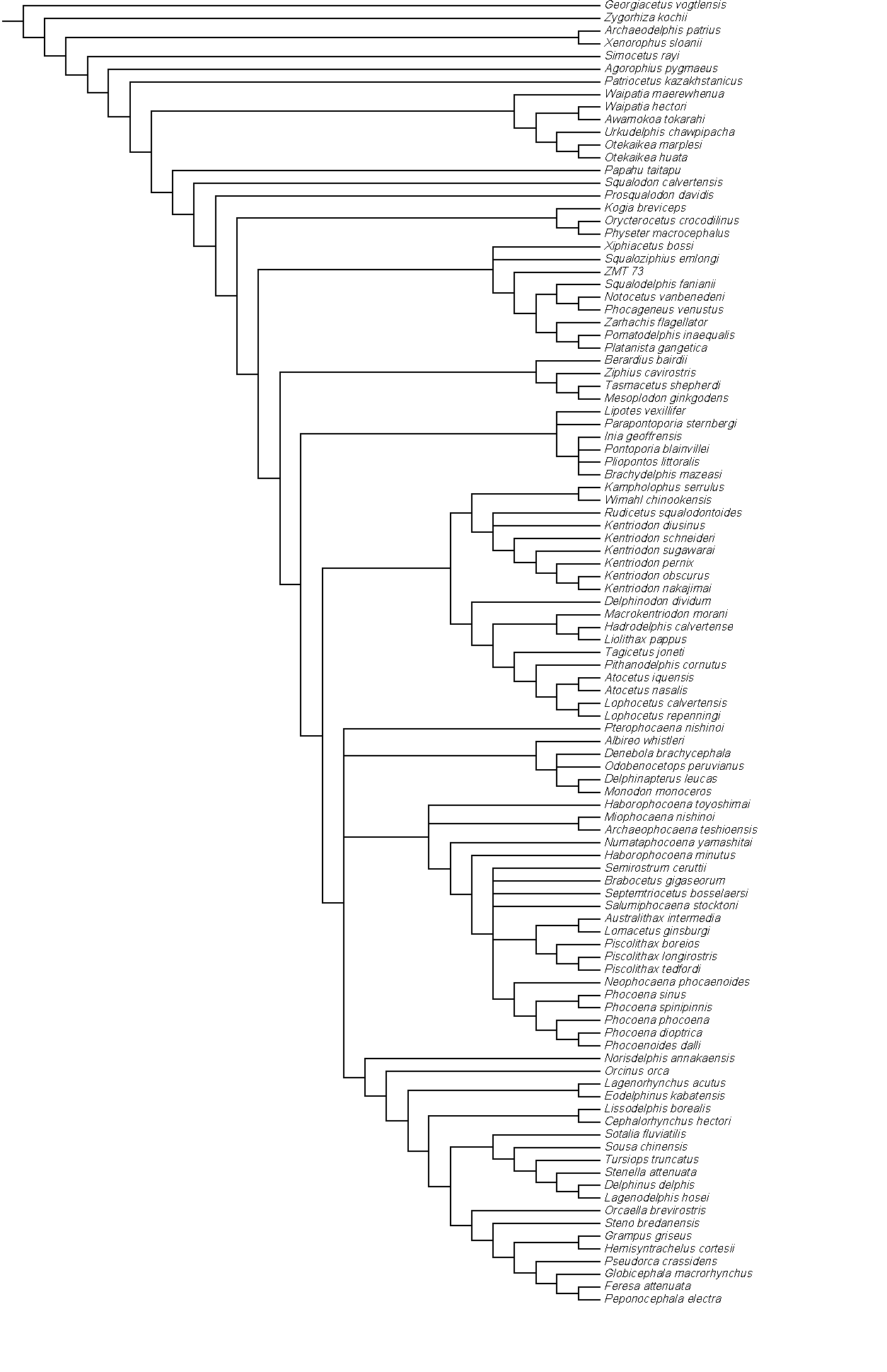
APPENDIX S1 (Figure S3). The strict consensus tree resulting from 256 most parsimonious trees with tree constraint by the molecular tree.

APPENDIX S2. Institutional abbreviations and list of taxa and specimens examined. Taxa and specimens in bold are examined for missing code, while all others were originally studied by and are listed in the Supporting Information File 1 of Lambert et al. (2017), or in the Supplementary Data 1 of Murakami et al (2012b).

**Institutional Abbreviations**

**AMNH**, American Museum of Natural History, New York, New York, USA; **CAS**, California Academy of Sciences, San Francisco, California, USA; **CMM**, Calvert Marine Solomons, Maryland, USA; **FCMM**, Colección de Referencia del Estado de Baja California, Ensenada, Baja California, México; **GMNH**, Gunma Museum of Natural History, Gunma, Japan; **HM**, Hokkaido Museum, Hokkaido, Japan; **HMNH**, Haboro Museum of Natural History, Hokkaido, Japan: **IGF**, Museo di Geologia e Paleontologia dell'Università di Firenze, Italy; **IGUP**, Museo di Geologia e Paleontologia of the Università di Padova, Padova, Italy; **IRSNB**, Institute Royal des Sciences Naturelles, Bruxelles, Belgium; **LACM**, Natural History Museum of Los Angeles County, California, USA; **MCZ**, Museum of Comparative Zoology, Harvard College, Cambridge, Massachusetts, USA; **MGGC**, Museo Geologico Giovanni Capellini dell'Università di Bologna, Bologna, Italy; **MGP**, Museo Geopaleontologico G. Capellini, Università di Bologna, Bologna, Italy; **MGPT**, Museo Geopaleontologico, Università di Torino, Torino, Italy; **MNHN**, Museúm National d’Histoire Naturelle, Paris, France; **MO**, Montañita/OloÂn collection, at Universidad Estatal Peninsula de Santa Elena (UPSE), Ecuador; **MPST**, Museo Palaeontologico di Salsomaggiore Terme; Salsomaggiore Terme, Italy; **MSNT**, Museo di Storia Naturale e del Territorio, Università di Pisa, Pisa, Italy; **NFL**, Numata Fossil Laboratory, Hokkaido, Japan; **NMHF**, Ninohe Museum of History and Folklore, Ninohe City, Iwate, Japan; **NMNS**, National Museum of Nature and Science, Tokyo, Japan; **NMNZ**, Museum of New Zealand Te Papa Tongarewa, Wellington, New Zealand; **NMV**, Nakagawa Museum of Natural History, Hokkaido, Japan; **OPCF**, Ocean Park Conservation Foundation, Hong Kong; **OU**, Geology Museum, University of Otago, Dunedin, New Zealand; **SDSNH**, San Diego Natural History Museum, San Diego, California, USA; **SMNK**, Staatliches Museum für Naturkunde, Karlsruhe, Germany; **UCMP**, University of California Museum of Paleontology, Berkley, California, USA; **UCMVZ**, Museum of Vertebrate Zoology, University of California, Berkley, California, USA; **UCR**, University of California at Riverside, Riverside, California, USA; **UF**, Florida Museum of Natural History, University of Florida, Gainesville, Florida, USA; **USNM**, United States National Museum of Natural History, Smithsonian Institution, Washington, DC, USA; **UWBM**, Burke Museum, University of Washington, Seattle, Washington, USA; **ZMT**, Fossil mammals catalogue, Canterbury Museum, Christchurch, New Zealand; **ZUCM**, Facultad de Humanidades y Ciencias, Montevideo, Uruguay.

**List of the Specimens**

*Georgiacetus vogtlensis* † GSM 350

***Zygorhiza kochii*** † NMNS-PV 17652 (cast of USNM 11962), USNM 4678, **4679**, **4680**, **4748**, **10855**, **11962**, **12975**, 16639

*Archaeodelphis patrius* † AMNH 18601, USNM 467596

*Xenorophus sloanii* † USNM 11049

*Agorophius pygmaeus* † LACM 143475, USNM 243980, 449561

*Patriocetus kazakhstanicus* † USNM 510829

*Orycterocetus crocodilinus* † USNM 14729, 14730, 22926, 22931, 22953, USNM 336585, 364222

*Physeter macrocephalus* NMNS-M 3539, 24818, 24819, 25991, MSNT UP 266, USNM A 35315, USNM 253051

*Kogia breviceps* AMNH 141 58, 36595, NMNS-M 24773

*Tasmacetus shepherdi* USNM 484878

*Berardius bairdii* NMNS-M 3535, 25987, 25988, 26427, 30129

*Ziphius cavirostris* AMNH 40015, 40016, NMNS-M 3537, 24291, 26423, 26426, 31331

*Mesoplodon ginkgodens* NMNS-M 8743, 24870, 24937, 35126

*Xiphiacetus bossi* † USNM 8842, 11867, 16581, 16890, 23086, 23216, 25670

*Simocetus rayi* † NMNS-PV 17780 (cast of USNM 256517)

*Lipotes vexillifer* AMNH 57333, USNM 218293

*Parapontoporia sternbergi* † LACM SDSNH 22633, 75060, 123658

*Inia geoffrensis* NMNS-M 24891, USNM A 49582, USNM 239667, 395614, 395415, 395602, 396166

*Pontoporia blainvillei* NMNS-M 24982, 36353, 36355, UCMVZ 148033, USNM 482707

*Pliopontos littoralis* † MNHN SAS 193, 931, 953

*Brachydelphis mazeasi* † MNHN PPI 121, 124, 230, PPI 233, XXX

***Atocetus iquensis* † NMNH PPI 113, 114, 115**

***Kentriodon pernix*** † IRSNB 372, **NMNS-PV 17784 (cast of USNM 10670)**, USNM 8060, **10670**

***Hadrodelphis calvertense*** † **CMM V 11**, USNM 170993, USNM 23408, USNM 182938

*Albireo whistleri* † UCMP 314589

***Denebola brachycephala* † UCR 21245**

***Delphinapterus leucas*** AMNH 10192, 180017, 212184, 212552, 34937, NMNS-M 5630, 34189, USNM A 7382, USNM 238105, MSNTUP 276, 277, 293

*Monodon monoceros* AMNH 193 16, LACM 72548, MSNTUP 274, 275, NMNS-M 33766, USNM A 22973, 22983, USNM 267957, 267958, 267961

***Odobenocetops peruvianus*** † **MNHN SAS 1613, 1614,** **USNM 488252**, LACM no catalogue number (cast of SMNK PAL 2491), NMNS-PV 20741 (cast of SMNK PAL 2491)

*Pterophocaena nishinoi* † NMV 7

*Australithax intermedia* † MNHN PPI 65

*Lomacetus ginsburgi* † MNHN PPI 104, USNM 452992

***Miophocaena nishinoi* † NMV 6**

***Archaeophocaena teshioensis* † NMV 56**

*Haborophocoena minutus* † SMAC 1388

*Haborophocoena toyoshimai* † HMNH 110 110-1

***Numataphocoena yamashitai* † NFL 7**

*Piscolithax boreios* † UCR 15975

*Piscolithax longirostris* † MNHN SAS 933, 934, 940

*Piscolithax tedfordi* † UCR 15972

***Semirostrum ceruttii* † SDSNH 65276**

***Brabocetus gigaseorum* † IRSNB M. 2171**

***Septemtriocetus bosselaersi* † IRSNB M.1928**

***Salumiphocaena stocktoni* † UCMP 34576**

***Phocoena sinus*** LACM 28259, **SDSNH 20688**, USNM 303308, 395722, 395723, 395892, 571431

***Phocoena spinipinnis*** UCMVZ 145780, **USNM 395379**, 395729, 395738, 395739, 395743, **395754**, 550782

***Phocoena dioptrica*** LACM 86042, USNM 571484, 57185, 57186, **ZUCM 850**

*Phocoena phocoena* NMNS-M 24648, 24902, 24902, 24958, 24960, 27391, 27393, 28367, USNM 571592, 571709, 571883, 572783

*Phocoenoides dalli* LACM 43473, NMNS-M 21382, 21383, 21384, 24679, 24684, 24686, 24687, 24688, 24689, 24693, 24944, 25177, 25233, 25942, USNM 276064, 286865, 504417, 504969

***Neophocaena phocaenoides*** NMNS-M 24659, 24660, 24945, 24946, 24947, 24949, 24950, 24953, 24964, 27005, 27885, 28371, 28371, 28436, 30114, **52646**, USNM 239990, 240001, 241503, 550473, 504910

***Lissodelphis borealis*** NMNS-M 24743, 24782, 24783, 24828, **24829**, 25182, 26628, 27724

***Cephalorhynchus hectori*** USNM A 84588, USNM 500864, 571649, **MSNTUP 292**, NMNZ MM 002607

*Lagenorhynchus acutus* USNM A 14279, 22942, USNM 504166, 504763, 571387, 571402

*Orcaella brevirostris* USNM 199743, 486170

*Orcinus orca* LACM 52479, 72577, NMNS-M 4510, 21262, 32458, 33459, USNM A 22068, USNM 219326, 239537, 550857

***Feresa attenuata*** NMNS-M 24591, 24593, 24594, 24595, 32546, **54968**, USNM 395177, 484995, 504917, 550389

*Globicephala macrorhynchus* NMNS-M 4511, 24603, 24604, 24802, 24810, 24609, USNM A 22571, 37261, USNM 504396

*Grampus griseus* NMNS-M 21400, 24664, 25981, 29490, 29524, 29544, USNM 504328, 55 0308, 571602, 572474

*Hemisyntrachelus cortesii* † IGF 1525V, 1545V, MB 8547 1COC1, 8548 1COC2, 8550 1COC4, 8551 1COC5, 8552 1COC 6, 8556, 1COC10, 8566 1COC20, 8567 1COC21, 8568 1COC22, MGPT PU13874, 13875, 13880, 13882, MPST A, B

***Peponocephala electra*** NMNS-M 21284, 21285, 24654, 24657, **34075**, USNM 504503, 504504

*Pseudorca crassidens* NMNS-M 24616, 24772, 29345, 32743, USNM 218360, 484982

*Delphinus delphis* NMNS-M 24646, 24647, 24698, 25180, 25263, 26350, 500134, 550750, USNM 482800

***Lagenodelphis hosei*** NMNS-M **24920**, 24921, 29574, 29575, USNM 571619

***Sousa chinensis*** LACM 72168, **OPCF SC97-31/05-B**, USNM 258859

***Stenella attenuata*** NMNS-M 23655, 24666, 24668, 24703, 24926, 24927, 25130, 25135, **25230**, 27415, USNM 261431, 487118

*Tursiops truncatus* NMNS-M 24635, 24788, 24618, 24702, 24738, 24907, 24923, 32763, USNM 572707

***Sotalia fluviatilis*** LACM 19594, 49708, 49709, USNM 253476, 571461, 571558

***Steno bredanensis*** NMNS-M 19770, 23622, 24637, 24639, 25197, 29467, **54920**, USNM A 49628, 49983, USNM 572802

*Eodelphinus kabatensis* † HM 68037

*Papahu taitapu* † OU 22066

***Waipatia hectori* † NMNZ Ma 653**

***Awamokoa tokarahi* † OU 22125**

*Waipatia maerewhenua* † OU 22095, USNM 508061

***Otekaikea marplesi* † OM GL 421**

***Otekaikea huata* † OU 22306**

*Squalodon calvertensis* † NMNS-PV 17787 (cast of USNM 10484), NMNS-PV 15396 (cast of USNM 206288), USNM 10484, 22902, 23537, 25910, USNM PV 175382

*Notocetus vanbenedeni* † AMNH 9485, 29060

***Squalodelphis fabianii* † IGUP 26134**

*Prosqualodon davidis* † AMNH 18601

*Squaloziphius emlongi* † USNM 181528

*Zarhachis flagellator* † NMNS-PV 17788 (cast of USNM 10485), USNM 10485, 10911, 16633

***Pomatodelphis inaequalis*** † **MCZ 4433**, UF 54000, USNM 187414

*Platanista gangetica* CAS 16340, NMNS-M 24853, 24857, 24859, 24861, 24862, 24936, USNM A 23456

*Phocageneus venustus* † USNM 21039

**ZMT 73 † ZMT 73**

***Urkudelphis chawpipacha* † MO 1**

***Lophocetus calvertensis* † NMNS-PV 17781 (cast of USNM 16314), USNM 16314**

***Lophocetus repenningi* † NMNS-PV 17783 (cast of USNM 23886), USNM 23886**

***Atocetus nasalis* † LACM 63335, 30093**

***Liolithax pappus* † NMNS-PV 17782 (cast of USNM 15985), USNM 15985**

***Delphinodon dividum* † USNM 7278**

***Kampholophos serrulus* † UCMP 36045**

***Macrokentriodon morani* † CMM-V-15, USNM 299694**

***Rudicetus squalodontoides* † MGGC 8599**

***Pithanodelphis cornutus* † IRSNB 3652-M.373**

***Tagicetus joneti* † IRSNB M.1892**

***Kentriodon obscurus* † LACM 21256, CAS 4347, 4349**

***Kentriodon diusinus* † FCMM 2387**

***Kentriodon schneideri* † USNM 323772**

***Wimahl chinookensis* † UWBM 88078**

***Kentriodon nakajimai* † GMNH PV 95, 1010, 3244, 3245**

***Norisdelphis annakaensis* † GMNH PV 3463**

***Kentriodon* *sugawarai* † NMHF 999**

APPENDIX S3. List of morphological characters and characters states used in phylogenetic analysis. Terminology generally follows that of the cladistic papers cited, which in a few cases does not agree with the recommended usage of Mead and Fordyce (2009). For each character, references are given for the past uses and definitions, with the relevant published character number given with a hatch # thus: Tanaka et al. (2017) #1.

**Rostrum, Dental, and Mandibular**

1. Length of rostrum as percent skull length: moderately long, 50–55% (0); long, 55–60% (1); very long, >60% (2); medium, 50–40% (3); very short, 40–35% (4) (Murakami et al. (2012a, 2012b) #1; modified from Arnold and Heinsohn, (1996) #8; Bianucci, (2005) #1; Lambert, (2008) #1; Tanaka and Fordyce, (2014, 2015) #1; Tanaka et al. (2017) #1).
2. Premaxillae transverse proportion: transversely inflated almost entire length of rostrum (0); flat almost entire length of the rostrum (1) (Murakami et al. (2012a, 2012b) #2; Tanaka and Fordyce, (2014, 2015) #2; Tanaka et al. (2017) #2).
3. Premaxillae mediolateral proportion: not compressed mediolaterally (0); compressed mediolaterally at anterior of rostrum (1) (Murakami et al. (2012a, 2012b) #3; Tanaka and Fordyce, (2014, 2015) #3; Tanaka et al. (2017) #3).
4. Premaxillae at apex of rostrum: with lateral margins parallel or diverging (0); narrowing (1) (Murakami et al. (2012a, 2012b) #4; modified from Bianucci, (2005) #2; Tanaka and Fordyce, (2014, 2015) #4; Tanaka et al. (2017) #4).
5. Maxilla length as percent rostrum length: short, <85%, tips of maxillae not reaching tip of rostrum, (0); long, >89%, tips of maxillae to within 10% of rostrum tip (1); same as state 1 except lack of alveoli (2) (Murakami et al. (2012a, 2012b) #5; modified from Lambert, (2005) #1; Tanaka and Fordyce, (2014, 2015) #5; Tanaka et al. (2017) #5).
6. Mesorostral groove: V-shaped or U-shaped opening (0); partially or completely filled in with vomer, becoming a solid rod of bone (1); absent (2) (Messenger and McGuire, (1998) #1429; Geisler and Sanders, (2003)#5; Geisler et al., (2011) (2012) #5; Murakami et al. (2012a, 2012b) #6; derived from Moore, (1968); Tanaka and Fordyce, (2014, 2015) #6; Tanaka et al. (2017) #6).
7. Mesorostral groove constricted posteriorly, anterior to the nares and behind the level of the antorbital notch, then rapidly diverging anteriorly: absent (0); present (1) (modified from Murakami et al. (2012b) #279; Tanaka and Fordyce, (2014, 2015) #7; Tanaka et al. (2017) #7).
8. Lateral margin of rostrum anterior to maxillary flange: concave (0); straight (1); convex (2); absent (3) (Murakami et al. (2012a, 2012b) #7; modified from Bianucci, (2005) #3; Tanaka and Fordyce, (2014, 2015) #8; Tanaka et al. (2017) #8).
9. Rostral constriction: absent (0); constriction anterior to antorbital notch (1); constriction anterior to maxillary flange (2) (Murakami et al. (2012a, 2012b) #8; modified from Muizon, (1984); Barnes, (1985b); Messenger and McGuire, (1998) #1424; Geisler and Sanders, (2003) #6; Geisler et al., (2012; 2011) #6; Tanaka and Fordyce, (2014, 2015) #9; Tanaka et al. (2017) #9).
10. Antorbital notch: absent or weakly developed (0); well developed (1) (Messenger and McGuire, (1998) #1426; Fajardo-Mellor et al., (2006) #6; Murakami et al. (2012a, 2012b) #9; Tanaka and Fordyce, (2014, 2015) #10; Tanaka et al. (2017) #10).
11. Width of premaxillae at mid-rostrum as percent greatest width of maxillae at level of postorbital processes: wide, >25% (0); medium, 25–15% (1); narrow, <15% (2) (Murakami et al. (2012a, 2012b) #10; modified from Aguirre-Fernandez et al., (2009) #4); Tanaka and Fordyce, (2014, 2015) #11; Tanaka et al. (2017) #11).
12. Width of rostrum at mid-length as percent greatest width of maxillae at level of postorbital processes: wide, >35% (0); medium, 35–30% (1); narrow, <30% (2) (Murakami et al. (2012a, 2012b) #11; modified from Aguirre-Fernandez et al., (2009) #6; Tanaka and Fordyce, (2014, 2015) #12; Tanaka et al. (2017) #12).
13. Width of rostrum at antorbital notch as percent greatest width of maxillae at level of postorbital processes: wide, >68% (0); medium, 68–45% (1); narrow, <45% (2) (Murakami et al. (2012a, 2012b) #12; modified from Geisler and Sanders, (2003) #7; Geisler et al., (2012; 2011) #7; Tanaka and Fordyce, (2014, 2015) #13; Tanaka et al. (2017) #13).
14. Posterior end of palatal surface of rostrum at the suture between palatine and maxilla (ordered): Concave to flat, depth of rostrum, measured as the dorsoventral distance from the level of the lateral edge of rostrum to the ventral-most part of rostrum, is < 8% the width of rostrum at antorbital notches (0); convex, depth between 11% and 25% the rostral width (1); highly convex, rostral depth > 27% the rostral width (2) (Lambert, et al, (2017) #19) The convexity of the rostrum is a part of characters included by Barnes (1985b) and Fordyce (1994).
15. Premaxillae in dorsal view: contacting along midline for less than half length of rostrum (0); widely separated by mesorostral groove in rostrum (1); narrowly separated by mesorostral groove in rostrum (2); contacting along midline for approximately half the entire length or more than of rostrum but not fused (3); contacting along midline for approximately half the entire length or more than of rostrum and partially fused (4); converging (either contacting and separating) in mid-rostrum (5) (modified from Murakami et al. (2012a, 2012b) #13; modified from Muizon, (1988); Fordyce, (1994) #52; Messenger and McGuire, (1998) #1405; Geisler and Sanders, (2003) (9); Geisler et al., (2011) (2012) #9; Tanaka and Fordyce, (2014, 2015) #14; Tanaka et al. (2017) #14).
16. Premaxilla in dorsal view: Portion adjacent to and anterior to nasal opening narrows or remains the same width anteriorly (0); widens at anterior end (1) (Lambert, et al, (2017) #8).
17. Suture between maxilla and premaxilla on rostrum (ordered): Suture fused along most of rostrum (0); anterior quarter of rostrum fused with remaining portions unfused (1); unfused along entire rostrum but articulation tight (2); suture is unfused and marked by a deep grooved (3) (modified from Fordyce, (1994); Messenger & McGuire, (1998); Lambert, et al, (2017) #11).
18. Posterior wall of antorbital notch: maxilla (0); lacrimal and jugal, or maxilla appeared in small area posterior to antorbital notch parallel with lacrimal and jugal (1); no notch but horizontal groove inferred to be for the facial nerve in the maxilla laterally on the face well above the margin of the rostrum (2) (Murakami et al. (2012a, 2012b) #15; modified from Geisler and Sanders, (2003) (15); Geisler et al., (2011) (2012) #15; Tanaka and Fordyce, (2014, 2015) #16; Tanaka et al. (2017) #16).
19. Anterior sinus fossa: absent (0); between anterior extremity of pterygoid sinus and posterior extremity of upper tooth row (1); between posterior extremity of upper tooth row and midpoint of rostrum (2); beyond midpoint of rostrum (3) (Murakami et al. (2012a, 2012b) #17; modified from Muizon, (1988); Barnes, (1990); Bianucci, (2005) #13; Arnold and Heinsohn, (1996) #21; Geisler and Sanders, (2003) #157; Aguirre-Fernandez et al., (2009) #18; Geisler et al., (2011) (2012) #157; derived from Fraser and Purves, (1960); Tanaka and Fordyce, (2014, 2015) #18; Tanaka et al. (2017) #17).

**Teeth**

1. Number of double-rooted teeth in maxilla: 6–8 (0); 0 (1) (modified from Geisler and Sanders, (2003) #23; Geisler et al., (2011) (2012) #23; Murakami et al. (2012a, 2012b) #18; Tanaka and Fordyce, (2014, 2015) #19; Tanaka et al. (2017) #18).
2. Tooth enamel: reticulating striae (0); smooth (1); nodular (2); absent (3) (Murakami et al. (2012a, 2012b) #20; modified from Messenger and McGuire, (1998) #1469; Geisler and Sanders, (2003) #26; Geisler et al., (2012; 2011) #26; derived from Zhou, (1982); Tanaka and Fordyce, (2014, 2015) #20; Tanaka et al. (2017) #19).
3. Teeth: heterodont and some teeth with denticle (0); conical, with or without accessory cusp (1); spatulate (2); laterally compressed (3) (Murakami et al. (2012a, 2012b) #21; modified from Heyning, (1989) #40, (1997) #72; Arnold and Heinsohn,(1996) #25; Messenger and McGuire, (1998) #1470; Geisler and Sanders, (2003) #27, (2012) #27; Lambert, (2008) #16; Geisler et al., (2011) #27; derived from Barnes, (1984a); Tanaka and Fordyce, (2014, 2015) #21; Tanaka et al. (2017) #20).
4. Upper anterior teeth: about same size as upper posterior teeth (0); greatly enlarged (1); clearly smaller than upper posterior teeth or absent (2) (modified from Murakami et al. (2012a, 2012b) #22; Tanaka and Fordyce, (2014, 2015) #22; Tanaka et al. (2017) #21).
5. Incisors relatively delicate and procumbent: no (0); yes (1) (Fordyce (1994) #42; Tanaka and Fordyce, (2014, 2015) #285; Tanaka et al. (2017) #22).
6. Crown of heterodont teeth: long (>10 mm) (0); short (<10mm) (1) (Fordyce (1994) #58; Tanaka and Fordyce, (2014, 2015) #286; Tanaka et al. (2017) #23).
7. Large incisor tusk: absent (0); present (1) (Tanaka and Fordyce, (2014, 2015) #287; Tanaka et al. (2017) #24).
8. Cheek teeth ectocingulum: present (0); absent (1) (Murakami et al. (2012a, 2012b) #23; modified from Geisler and Sanders, (2003) #31; Geisler et al., (2012; 2011) #31; Tanaka and Fordyce, (2014, 2015) #23; Tanaka et al. (2017) #25).
9. Cheek teeth entocingulum: present (0); absent (1) (Geisler and Sanders, (2003) #32; Geisler et al., (2012; 2011) #32; Murakami et al. (2012a, 2012b) #24; Tanaka and Fordyce, (2014, 2015) #24; Tanaka et al. (2017) #26).
10. Large diastemata between posterior buccal teeth: Absent (0); present (1) (Lambert, et al, (2017) #26).
11. Lower anterior mandibular teeth: Conical (0); spatulate (1); laterally compressed (2) (Messenger & McGuire, (1998); Lambert, et al, (2017) #28). Heyning (1989) described state “1” and Moore (1968) described state “2”.
12. Swelling on lingual side of posterior lower teeth: Present (0); absent (1) (Flower, (1867); Messenger & McGuire, (1998); Lambert, et al, (2017) #30).
13. Posterior buccal teeth (ordered): High peg-shaped teeth, crown base is < 70% the crown height (0); nearly an equilateral triangle, crown base is between 100% to 148% the crown height (1); wide low teeth, crown base is > 180% the crown height (2) (Lambert, et al, (2017) #31).
14. Buccal teeth: Bear accessory cusps (0); cusps absent (1) (Kellogg, (1923); Lambert, et al, (2017) #34).
15. Posterior lower teeth (ordered): Apex not recurved (0); apex slightly recurved lingually (1); apex strongly recurved lingually (2) (Lambert, et al, (2017) #315).
16. Greatest diameter of largest functional tooth as percent of greatest width of maxillae at the level of the postorbital processes: large, >5% (0); medium, 5–3% (1); small, <3% (2) (Murakami et al. (2012a, 2012b) #25; modified from Aguirre-Fernandez et al., (2009) #15; Tanaka and Fordyce, (2014, 2015) #25; Tanaka et al. (2017) #2).

**Mandible**

1. Anterior mandibular teeth: deeply rooted, root >50% of tooth (0); not deeply rooted, root <50% of tooth (1) (Messenger and McGuire, (1998) #1471; Geisler and Sanders, (2003) #28; Geisler et al., (2012; 2011) #28; Murakami et al. (2012a, 2012b) #26; derived from Flower, (1872); Moore, (1968); Tanaka and Fordyce, (2014, 2015) #26; Tanaka et al. (2017) #28).
2. Anterior-most mandibular teeth (ordered): Oriented anteriorly (0); vertical (1); inclined posteriorly (2) (Moore, (1968); Messenger & McGuire, (1998); Lambert, et al, (2017) #36).
3. Anterior-most mandibular "tooth": about same size as posterior teeth (0); smaller than posterior teeth (1); greatly enlarged (2); forming a tusk (3) (Murakami et al. (2012a, 2012b) #27; modified from Muizon, (1991); Geisler and Sanders, (2003) #36; Messenger and McGuire, (1998) #1477; Geisler et al., (2012; 2011) #36; derived from Flower, (1872); Tanaka and Fordyce, (2014, 2015) #27; Tanaka et al. (2017) #29).
4. Number of teeth in mandible: 16–11 (0); 9–8 (1); 2 (2); 1 (3); 17–23 (4); 24–27 (5); 28–39 (6); >40 (7) (Murakami et al. (2012a) #28; modified from Messenger and McGuire, (1998) #1468; Geisler and Sanders, (2003) #37; Geisler et al., (2012; 2011) #37; Tanaka and Fordyce, (2014, 2015) #28; Tanaka et al. (2017) #30).
5. Length of mandibular symphysis as percent of mandible length: long, >20% (0); short, <20% (1) (Murakami et al. (2012a, 2012b) #29; modified from Messenger and McGuire, (1998) #1465; Arnold and Heinsohn,(1996) #7; Bianucci, (2005) #26; Tanaka and Fordyce, (2014, 2015) #29; Tanaka et al. (2017) #31).
6. Mandibular symphysis: sutured but unfused (0); fused (1) (Fordyce, (1994) #5; Messenger and McGuire, (1998) #1466; Geisler and Sanders, (2003) #40; Geisler et al., (2011) (2012) #40; Murakami et al. (2012a, 2012b) #30; derived from Flower, (1885); Tanaka and Fordyce, (2014, 2015) #30; Tanaka et al. (2017) #32).
7. Longitudinal groove on underside of mandibles: absent (0); present (1) (Geisler and Sanders, (2003) #41; Geisler et al., (2012; 2011) #41; Murakami et al. (2012a, 2012b) #31; derived from Miller, (1923); Tanaka and Fordyce, (2014, 2015) #31; Tanaka et al. (2017) #33).
8. Mandible: bowed medially (0); straight (1); slightly bowed laterally (2) (Sanders and Barnes, 2002; Geisler and Sanders, (2003) #42; Geisler et al., (2011) (2012) #42; Murakami et al. (2012a, 2012b) #32; derived from Miller, (1923); Tanaka and Fordyce, (2014, 2015) #32; Tanaka et al. (2017) #34).
9. Dorsal surface of condyle: Elevated above dorsal edge of the rest of mandible, not counting coronoid process (0); at same level as rest of mandible (1) (Sanders & Barnes, (2002); Lambert, et al, (2017) #46).
10. Elevation of coronoid process: very high (0); moderate (1); low (2) (Murakami et al. (2012a, 2012b) #33; modified from Geisler and Sanders, (2003) #44; Bianucci, (2005) #27; Geisler et al, (2012; 2011) #44; Tanaka and Fordyce, (2014, 2015) #33; Tanaka et al. (2017) #35).

**Orbit**

1. Supraorbital processes of frontal: Are horizontal or gradually slope lateroventrally away from vertex of skull (0); abruptly depressed at base to a level noticeably below that of dorsal surface of interorbital region (1); slope laterodorsally away from vertex (2) (Miller, (1923); Messenger & McGuire, (1998); Lambert, et al, (2017) #47).
2. Dorsal edge of orbit relative to lateral edge of rostrum (ordered): Below the level of the edge of rostrum (0); orbit low, either in line with edge of rostrum or slightly above it, height of orbit < 46% the height of rostral base, both heights measured relative to the lateral edge of rostrum (1); orbit low, height of dorsal edge of orbit between 50% and 92% the rostral height (2); orbit high, height between 100% and 128% the rostral height (3); orbit elevated well above rostrum, orbital height > 163% the rostral height (4) (Lambert, et al, (2017) #48).
3. Antorbital process of maxilla in dorsal view: triangular (0); robust and globose or rectilinear (1); absent (2) (Bianucci, (2005) #4; Murakami et al. (2012a, 2012b) #34; Tanaka and Fordyce, (2014, 2015) #34; Tanaka et al. (2017) #36).
4. Posterior region of rostral edge (ordered): Lateral margin is straight or gently concave with skull in dorsal view (0); slightly bowed outward causing a V-shaped antorbital notch (1); bowed far outward forming a deep U-shaped antorbital notch (2); lateral margin of maxilla nearly contacts lacrimal and jugal resulting in the opening of the notch being a narrow slit (3) (Lambert, et al, (2017) #12).
5. Anterior edge of the supraorbital process (ordered): Oriented anteromedially (0); oriented slightly anterolaterally, forms an angle < 30° with sagittal plane (1); oriented anterolaterally, forms an angle between 35° and 60° (2); oriented anterolaterally or laterally, forms and angle between 68° and 90° degrees (3); oriented posterolaterally, forms an angle between 107° and 120° (4); oriented posterolaterally, forms an angle > 142° (5) (Lambert, et al, (2017) #50) The anterior edge of the supraorbital process participates in the formation of the antorbital notch. For this character supraorbital process includes frontal, lacrimal, and/or jugal, whichever bone forms the posterolateral wall of the notch. An antorbital notch opening anteriorly was listed by Barnes (1990) as a synapomorphy of Odontoceti.
6. Ratio of length of antorbital process of lacrimal to length of the orbit: <0.6 (0); ≥0.6 (1) (Murakami et al. (2012a, 2012b) #36; Tanaka and Fordyce, (2014, 2015) #36; Tanaka et al. (2017) #38).
7. Lacrimal: wrapping around anterior edge of supraorbital process of frontal and slightly overlying its anterior end (0); appearing dorsally and forming most of antorbital process (1); appearing dorsally but not prominently in antorbital process (2); restricted to below the supraorbital process of maxilla (3) (Murakami et al. (2012a, 2012b) #37; modified from Geisler and Sanders, (2003) #51; Geisler et al., (2012; 2011) #51; derived from Kellogg, (1923); Miller, (1923); Tanaka and Fordyce, (2014, 2015) #37; Tanaka et al. (2017) #39).
8. Lacrimal foramen or groove: present (0); absent (1) (Geisler and Sanders, (2003) #52; Geisler et al., (2012; 2011) #52; Murakami et al. (2012a, 2012b) #38; Tanaka and Fordyce, (2014, 2015) #38; Tanaka et al. (2017) #40).
9. Lacrimal and jugal fusion: separated (0); fused (1) (Heyning, (1989) #7, (1997) #39; Geisler and Sanders, (2003) #53; Geisler et al., (2012; 2011) #53; Murakami et al. (2012a, 2012b) #39; derived from Flower, (1868); Schulte, (1917); Miller, (1923); Tanaka and Fordyce, (2014, 2015) #39; Tanaka et al. (2017) #41).
10. Lacrimal and jugal contact: contacting each other externally (0); lacrimal excluded from edge of skull, jugal directly contacting anterior edge of frontal (1) (Geisler and Sanders, (2003) #54; Geisler et al., (2012; 2011) #54; Murakami et al. (2012a, 2012b) #40; derived from Miller, (1923); Tanaka and Fordyce, (2014, 2015) #40; Tanaka et al. (2017) #42).
11. Jugal: thick and sturdy (0); thin, splint, or absent (1) (Geisler and Sanders, (2003) #56; Lambert, (2005) #21; Geisler et al., (2012; 2011) #56; Murakami et al. (2012a, 2012b) #41; derived from Miller, (1923); Barnes, (1990); Tanaka and Fordyce, (2014, 2015) #41; Tanaka et al. (2017) #43).
12. Combined anteroposterior length of the lacrimal and jugal exposure that is posterior to antorbital notch: with skull in ventral view, exposure is small and combined length forms <50% of anteroposterior distance from antorbital notch to postorbital ridge (0); intermediate, forms between 50 and 62% of that distance (1); large, forms between 62 and 69% that distance (2); very large, forms >69% of that distance (3) (Murakami et al. in (2012a, 2012b) #42; modified from Geisler and Sanders, (2003) #55; Geisler et al., (2012; 2011) #55; Tanaka and Fordyce, (2014, 2015) #42; Tanaka et al. (2017) #44).
13. Dorsolateral edge of internal opening of infraorbital foramen: formed by maxilla (0); formed by maxilla and lacrimal and/or jugal (l); formed by lacrimal and/or jugal (2); formed by frontal (3) (Geisler and Sanders, (2003) #57; Geisler et al., (2011) (2012) #57; Murakami et al. (2012a, 2012b) #43; derived from Miller, (1923); Tanaka and Fordyce, (2014, 2015) #43; Tanaka et al. (2017) #45).
14. Ventromedial edge of internal opening of infraorbital foramen: formed by maxilla (0); formed by maxilla and palatine and/or pterygoid (1); formed by palatine and/or pterygoid (2) (Geisler and Sanders, (2003) #58; Geisler et al., (2012; 2011) #58; Murakami et al. (2012a, 2012b) #44; derived from Miller, (1923); Tanaka and Fordyce, (2014, 2015) #44; Tanaka et al. (2017) #46).
15. Maxillary tuberosity: present (0); absent (1) (Geisler and Sanders, (2003) #59; Geisler et al., (2011) #59, (2012) #59; modified from Murakami et al. (2012a) #45; derived from Miller, (1923); Tanaka and Fordyce, (2014, 2015) #45; Tanaka et al. (2017) #47).
16. Direction of apex of postorbital process of frontal: projected posterolaterally and slightly ventrally (0); directed ventrally (1); not clear because of extremely reduced process (2) (modified from Murakami et al. (2012a, 2012b) #46; Geisler and Sanders, (2003) #61; Geisler et al., (2012; 2011) #61; Tanaka and Fordyce, (2014, 2015) #46; Tanaka et al. (2017) #48).
17. Shape of postorbital process of frontal: robust, blunt descending posteriorly (0); pointed, attenuated, or acute triangular (1); triangular, trapezoidal, or an anteroposteriorly widened falciform (2); dorsoventrally long falciform (3) (modified from Murakami et al. (2012a, 2012b) #47; Tanaka and Fordyce, (2014, 2015) #47; Tanaka et al. (2017) #49).
18. Frontal-maxilla suture angled posterodorsally at an angle of 50–70° (±) from axis of rostrum, with lateral exposure of frontal thickening posteriorly: absent (0); present (1) (Geisler and Sanders, (2003) #48; Geisler et al., (2011) #48, (2012) #48; Murakami et al. (2012a, 2012b) #48; derived from Miller, (1923); Tanaka and Fordyce, (2014, 2015) #48; Tanaka et al. (2017) #50).
19. Infratemporal crest (Postorbital ridge): Present, forms well-defined curved ridge on posterior edge of sulcus for optic nerve (0); no well-defined ridge, region is gently convex (1) (Lambert, et al, (2017) #63).

**Facial Region**

1. Anterior dorsal infraorbital foramina: one (0); two (1); three or more (2) (Murakami et al. (2012a, 2012b) #49; modified from Barnes, (1984b); Geisler and Sanders, (2003) #64; Geisler et al., (2011) #64, (2012) #64; Tanaka and Fordyce, (2014, 2015) #49; Tanaka et al. (2017) #51).
2. Rostral basin: absent or poorly defined (0); present, situated medial to antorbital notch and anterior to supraorbital process of frontal (1) (Geisler and Sanders, (2003) #65; Geisler et al., (2011) #65, (2012) #65; Murakami et al. (2012a, 2012b) #50; Tanaka and Fordyce, (2014, 2015) #50; Tanaka et al. (2017) #52).
3. Width of premaxillae at antorbital notches as percent width of rostrum at antorbital notch: narrow, <49% (0); moderate, 50–64% (1); wide, >65% (2); antorbital notch absent (3) (Geisler and Sanders, (2003) #66; Geisler et al., (2011)#66, (2012) #66; modified from Murakami et al. (2012a, 2012b) #51; Tanaka and Fordyce, (2014, 2015) #51; Tanaka et al. (2017) #53).
4. Premaxillae immediately anterior to external bony nares (ordered): Widely separate with skull in dorsal view, gap between medial edges of premaxillae > 63% the maximum width of external bony nares (0); narrow separation, gap between premaxillae between 56% and 32% the width of external nares (1); separation absent or nearly so, gap < 28% the nares width (2) (Lambert, et al, (2017) #68).
5. Premaxillary foramina (ordered): Absent (0); present and one on right side (1); two on right side (2); three on right side (3) (modified from Barnes, (1990); Lambert, et al, (2017) #70).
6. Size of premaxillary foramen: right and left subequal (0); right much larger than left (1); premaxillary foramen absent (2) (Messenger and McGuire, (1998) #1415; Murakami et al. (2012a, 2012b) #53; modified from Geisler and Sanders, (2003) #70; Geisler et al., (2011) #70, (2012) #70; Tanaka and Fordyce, (2014, 2015) #53; Tanaka et al. (2017) #54).
7. Position of premaxillary foramen: anterior of antorbital notch and anterior edge of supraorbital process (0); approximately medial to or posterior to antorbital notch region (1); premaxillary foramen absent (2) (Geisler and Sanders, (2003) #71; Geisler et al., (2011) #71, (2012) #71; Murakami et al. (2012a, 2012b) #54; Tanaka and Fordyce, (2014, 2015) #54; Tanaka et al. (2017) #55).
8. Premaxillary foramen locating: medial (0); midpoint to lateral (1) absent (2) (modified from Murakami et al. (Murakami et al., 2014): Murakami et al. (2012b) #280; Tanaka and Fordyce, (2014, 2015) #55; Tanaka et al. (2017) #56).
9. Lateral margin of the right premaxilla posterior to premaxillary foramen: widen posteriorly (0); straight (1) (Murakami et al. (2012b) #281; Tanaka and Fordyce, (2014, 2015) #56; Tanaka et al. (2017) #57).
10. Posterolateral sulcus: deep (0); shallow or absent (1); presence of additional posterolateral sulcus (longitudinal striation) (2) (Murakami et al. (2012a, 2012b) #55; modified from Muizon, (1984, 1988); Lambert, (2008) #6; Geisler and Sanders, (2003) #72; Geisler et al. (2011) (2012) #72; Tanaka and Fordyce, (2014, 2015) #57; Tanaka et al. (2017) #58).
11. Posterolateral sulcus from premaxillary foramen (ordered): Sulcus very short or absent (0); present and short (1); present and extends to level equivalent to middle of nasal openings (2) (modified from Muizon, (1988); Lambert, et al, (2017) #73).
12. Posterior projections of premaxillae: both premaxillae extending posterior to anterior tip of nasals (0); both premaxillae extending posterior to nasals (1); only right premaxillae extended posterior to nasal (2); neither premaxillae extending posterior to external nares, and narrow posterior end of premaxillae adjacent to external nares (3); neither premaxillae extending beyond external nares, and premaxillae displaced laterally by medial projection of maxilla (4); only right premaxilla extending beyond or in line with anterior-most portion of nasals (5) (Murakami et al. (2012a, 2012b) #76; modified from Muizon (1984); Barnes, (1985a); Heyning, (1989) #39, 42, (1997) #63, 71, 74; Arnold and Heinsohn,(1996) #35; Messenger and McGuire, (1998) #1407, 1408; Fajardo-Mellor et al., (2006) #3; Lambert, (2008) #5; Fordyce, (1994) #27; Tanaka and Fordyce, (2014, 2015) #58; Tanaka et al. (2017) #59).
13. Posteriormost end of nasal process of premaxilla (ordered): Located far anterior to anterior edge of supraorbital process/antorbital notch (0); just anterior to or in a transverse line with anterior edge of supraorbital process of the frontal (1); in line with anterior half of supraorbital process of frontal or halfway point, anteroposteriorly, of supraorbital process (2); in line with posterior half of supraorbital process or postorbital process of frontal (3); in line with gap between postorbital process and anterior tip of zygomatic process of the squamosal or in line with anterior tip of the latter process (4); in line with space between anterior tip of zygomatic process of squamosal and anterior edge of floor of the squamosal fossa or in line with anterior edge of floor of the squamosal fossa (5); located posterior to anterior edge of floor of the squamosal fossa (6) (Lambert, et al, (2017) #75).
14. Maxillary foramen (posteriormost dorsal infraorbital foramen): Absent (0); present and one, situated over supraorbital process of frontal (1); two (2); foramina absent because roof of canal that carries posterior branches of internal maxillary artery and the maxillary division of infraorbital nerve is unossified (3) (modified from Barnes, (1990); Lambert, et al, (2017) #76). The maxillary foramen (or foramina) is distinct from the anterior cluster of dorsal infraorbital foramina.
15. Posteriormost edge of the ascending process of maxilla (ordered): Situated well anterior to anterior edge of orbit (0); in transverse line with anterior half of supraorbital process of frontal or in line with the halfway point, anteroposteriorly, of supraorbital process (1); in line with posterior half of supraorbital process or in line with postorbital process of frontal (2); in line with gap between postorbital process and the anterior tip of zygomatic process of squamosal or in line with anterior tip of the latter process (3); in line with space between anterior tip of zygomatic process of squamosal and anterior edge of floor of squamosal fossa or in line with anterior edge of the floor of squamosal fossa (4); posterior to anterior edge of floor of squamosal fossa (5) (Lambert, et al, (2017) #78).
16. A posterior dorsal infraorbital foramen placed posteromedially, near posterior extremity of premaxilla: absent (0); present (1) (Fordyce, (1994) #62; Lambert, (2005) #13; Murakami et al. (2012a, 2012b) #58; Tanaka and Fordyce, (2014, 2015) #60; Tanaka et al. (2017) #60).
17. Premaxillary sac fossae: absent (0); present (1) (Messenger and McGuire, (1998) #1411; Lambert, (2005) #4; Murakami et al. (2012a, 2012b) #59; Tanaka and Fordyce, (2014, 2015) #61; Tanaka et al. (2017) #61).
18. Maxilla on dorsal surface of skull: does not contact supraoccipital posteriorly, maxilla separated by frontal and/or parietal (0); contact present (1) (Geisler and Sanders, (2003) #129; Geisler et al., (2011) #129, (2012) #129, modified from Muizon, (1991) (1994); Murakami et al., (2012a, 2012b) #60; Tanaka and Fordyce, (2014, 2015) #62; Tanaka et al. (2017) #62).
19. Maxillae at anterior edge of supraorbital processes: abutting anterior edge of supraorbital processes of frontals (0); covering partially or almost completely surface of supraorbital processes (1) (Murakami et al. (2012a, 2012b) #61; modified from Fordyce, (1994) #3; Messenger and McGuire, (1998) #1419; Geisler and Sanders, (2003) #76; Geisler et al., (2011) #76, (2012) #76; derived from Miller, (1923); Tanaka and Fordyce, (2014, 2015) #63; Tanaka et al. (2017) #63).
20. Anterolateral corner of maxilla overlying supraorbital process of frontal: thin and equal in thickness to parts posteromedial (0); thickened with thinner maxilla in posteromedial direction (1) (Geisler and Sanders, (2003) #78; Geisler et al., (2011) #78, (2012) #78; Murakami et al. (2012a, 2012b) #62; Tanaka and Fordyce, (2014, 2015) #64; Tanaka et al. (2017) #64).
21. Pneumatic maxillary crest overhanging medially: absent (0); present (1) (Zhou, (1982); Heyning, (1989) #26, (1997) #58; Fordyce, (1994) #66; Arnold and Heinsohn,(1996) #14; Messenger and McGuire, (1998) #1421; Murakami et al. (2012a, 2012b) #63; Tanaka and Fordyce, (2014, 2015) #65; Tanaka et al. (2017) #65).
22. Maxillary crest on supraorbital process of maxilla: longitudinal ridges absent except at lateral edge of antorbital process (0); presence of longitudinal ridge except at lateral edge of antorbital process (1); longitudinal ridge present and joined with maxillary flange (2); presence of transversely compressed high crest, except at lateral edge of antorbital process (3); absent (4) (Murakami et al. (2012a, 2012b) #64; modified from Muizon, (1984) (1987); Barnes, (1985b); Messenger and McGuire, (1998) #1420; Geisler and Sanders, (2003) #79; Geisler et al., (2011) #79, (2012) #79; derived from Miller, (1923); Tanaka and Fordyce, (2014, 2015) #66; Tanaka et al. (2017) #66).
23. Anterior edge of nasals: anterior to, or in line with, anterior edges of supraorbital processes of frontals (0); posterior to anterior edges of supraorbital processes of frontals (1) (Murakami et al. (2012a, 2012b) #65; modified from Geisler and Sanders, (2003) #80; Geisler et al., (2011) #80, (2012) #80; Tanaka and Fordyce, (2014, 2015) #67; Tanaka et al. (2017) #67).
24. Premaxillae in dorsal view: separated anterior to bony nares, exposing mesethmoid (0); joined premaxillae (or maxillae) closing at least posterior part of mesorostral groove (1) (Lambert, (2005) #3; Murakami et al. (2012a, 2012b) #66; Tanaka and Fordyce, (2014, 2015) #68; Tanaka et al. (2017) #68).
25. Anterior edge of bony nares: inverted V-shaped, premaxillae gradually converging anteriorly to midline (0); inverted U-shaped, premaxillae abruptly converging anteriorly to midline (1) (Muizon, (1988); Geisler and Sanders, (2003) #81; Geisler et al., (2011) #81, (2012) #81; Murakami et al. (2012a, 2012b) #67; Tanaka and Fordyce, (2014, 2015) #69; Tanaka et al. (2017) #69).
26. Fossa for inferior vestibule on maxilla lateral to external nares or lateral to premaxilla: absent (0); present (1) (Muizon, (1988); Murakami et al. (2012a, 2012b) #68; derived from Curry, (1992); Tanaka and Fordyce, (2014, 2015) #70; Tanaka et al. (2017) #70).
27. Maxillary intrusion, anterior to external nares and encroaching the posteromedial or medial face of each premaxilla: absent (0); maxilla visible within opened mesorostral canal as small exposure medially (1); exposure of maxilla reaches dorsally to level of premaxilla and forms a square, rectangular to triangular plate (2); exposure of maxilla reaches dorsally and forms a small subcircular to polygonal ossicle (3) (Muizon, (1984) (1988); Arnold and Heinsohn,(1996) #24; Messenger and McGuire, (1998) #1422; Murakami et al. (2012a, 2012b) #69; Tanaka and Fordyce, (2014, 2015) #71; Tanaka et al. (2017) #71).
28. Premaxillary crest or posterior maxillary crest adjacent to nasal: absent (0); present (1) (transverse premaxillary crest, sensu Lambert, (2005) #6; Murakami et al. (2012a, 2012b) #70; Tanaka and Fordyce, (2014, 2015) #72; Tanaka et al. (2017) #72).
29. Premaxilla: not overhanging itself or maxilla laterally (0); overhanging itself or maxilla laterally, from anterior to midpoint of external nares (1) (Murakami et al. (2012a, 2012b) #71; Tanaka and Fordyce, (2014, 2015) #73; Tanaka et al. (2017) #73).
30. Premaxillary sac fossa: smooth (0); rugose (1) (Messenger and McGuire, (1998) #1551; Murakami et al. (2012a, 2012b) #72; Tanaka and Fordyce, (2014, 2015) #74; Tanaka et al. (2017) #74).
31. Right premaxilla (ordered): Posterior edge approximately in line with posterior edge of left premaxilla (0); right premaxilla extended distinctly farther than left (1); right extended much farther than left (2) (modified from Barnes, (1990); Messenger & McGuire, (1998); Lambert, et al, (2017) #85).
32. Transverse width of right premaxilla immediately anterior to external bony nares (ordered): Distinctly narrower than left premaxilla (0); subequal, width of right premaxilla within 10% of the width of left premaxilla (1); right wider, width is between 130% and 145% the width of the left (2); right much wider, width > 167% the width of the left (3) (Lambert, et al, (2017) #86).
33. Ratio of greatest width of premaxillae to greatest width of maxillae at level of postorbital processes: ≥0.50 (0); 0.49–0.38 (1); <0.38 (2) (Murakami et al. (2012a, 2012b) #74; Tanaka and Fordyce, (2014, 2015) #76; Tanaka et al. (2017) #76).
34. Premaxillary eminence: absent (0); present but low (1); present and high (2) (Lambert, (2008) #4; Murakami et al. (2012a, 2012b) #75; modified from Muizon, (1984); Barnes, (1985a); Heyning, (1989) #36, (1997) #68; Arnold and Heinsohn,(1996) #12; Messenger and McGuire, (1998) #1410; Geisler and Sanders, (2003); #68; Fajardo-Mellor et al., (2006) #2; Geisler et al., (2011) #68, (2012) #69; derived from Flower, (1867); Noble and Fraser, (1971); Tanaka and Fordyce, (2014, 2015) #77; Tanaka et al. (2017) #77).
35. Intra-premaxillary foramen on posterior dorsal surface of skull, which is bounded by premaxilla and maxilla: absent (0); present (1) (Tanaka and Fordyce, (2014, 2015) #279; Tanaka et al. (2017) #78).
36. Right premaxilla: portion anterior to nasal opening wider than portion posterior to opening, with nasal septum angled anteriorly and to left (0); portion posterior to nasal opening wider than portion anterior to opening, with nasal septum angled anteriorly and to right (1) (modified from Geisler and Sanders, (2003); #86; Geisler et al., (2011) #86, (2012) #86; Murakami et al. (2012a, 2012b) #77; Tanaka and Fordyce, (2014, 2015) #78; Tanaka et al. (2017) #79).
37. Left bony naris: same size or slightly larger than right bony naris (0); at least twice the size of right bony naris (1) (Barnes, (1990); Geisler and Sanders, (2003) #87; Geisler et al., (2011) #87, (2012) #87; Murakami et al. (2012a, 2012b) #78; Tanaka and Fordyce, (2014, 2015) #79; Tanaka et al. (2017) #80).
38. Supracranial basin: absent (0); present (1) (Heyning, (1989) #8, (1997) #40; Fordyce, (1994) #18; Messenger and McGuire, (1998) #1400; Geisler and Sanders, (2003) #88; Lambert, (2005) #10; Geisler et al., (2011) #88, (2012) #88; Murakami et al. (2012a, 2012b) #79; Tanaka and Fordyce, (2014, 2015) #80; Tanaka et al. (2017) #81).
39. Proximal ethmoid region: not visible in dorsal view, roofed over by nasals (0); exposed dorsally (1) (Messenger and McGuire, (1998) #1455; Geisler and Sanders, (2003) #92; Geisler et al., (2011) #92, (2012) #92; Murakami et al. (2012a, 2012b) #80; derived from Miller, (1923); Tanaka and Fordyce, (2014, 2015) #81; Tanaka et al. (2017) #82).
40. Angle of premaxillae anterior to external bony nares, skull in lateral view (ordered): Low angle, premaxillae form an angle < 28° with the lateral edge of rostrum (0); intermediate angle, form an angle between 30° and 40° (1); high angle, form an angle > 45° (2) (modified from Moore, (1968); Lambert, et al, (2017) #91).
41. Mesethmoid: not expanded posterodorsally (0); extended posterodorsally but narrow (1); expanded posterodorsally and visible in lateral view (2) (Murakami et al. (2012a, 2012b) #81; modified from Muizon, (1984, 1988); Messenger and McGuire, (1998) #1454; Bianucci, (2005) #9; Tanaka and Fordyce, (2014, 2015) #82; Tanaka et al. (2017) #83).
42. Dorsal margin of mesethmoid (ordered): Below level of adjacent premaxilla (0); flush with or nearly flush with premaxilla (1); distinctly above level of adjacent premaxilla (2) (Lambert, et al, (2017) #306).
43. Maxillae (ordered): In region anterior to nasal openings, maxillae are exposed lateral to premaxillae (0); maxillae are exposed at posterior end of roof of mesorostral gutter, medial to the premaxillae and nearly converge on midline (1); same as 1 except maxilla also exposed on anterior edge of external bony nares (2) (Muizon, (1988); Lambert, et al, (2017) #83).
44. Mesethmoid: Forms T-shaped bone with median plate separating right and left nasal passages, not all of the dorsal part is divided by median plate (0); bears expanded posterodorsal plate which is not divided by median plate, median plate situated more ventrally (1) (modified from Muizon, (1984); Muizon, (1988); Lambert, et al, (2017) #94). This bone may include parts of the cribriform and perpendicular plates of the ethmoid.

**Vertex and Area Adjacent to the Nares**

1. Position of inflection of premaxilla (ordered): In transverse line with P1 (0); in line with P2 or about 18% of total rostral length towards anterior edge of rostrum (1); just anterior to or in line with anterior edge of supraorbital process of frontal (2); in line with anterior half of supraorbital process of frontal or in line with the halfway point, anteroposteriorly, of supraorbital process (3); in line with posterior half of supraorbital process or in line with postorbital process of frontal (4); in line with gap between postorbital process and the anterior tip of zygomatic process of squamosal or in line with the anterior tip of the latter process, space is absent in some taxa (5); in line with space between the anterior tip of zygomatic process of squamosal and anterior edge of the floor of squamosal fossa or in line with anterior edge of the floor of squamosal fossa (6); posterior to anterior edge of the floor of squamosal fossa (7) (Lambert, et al, (2017) #109) The position of the inflection cannot be scored for taxa that lack one.
2. Inflections of ascending processes of premaxillae: gradual (0); vertical (1) (Geisler and Sanders, (2003) #107; Geisler et al., (2011) #107, (2012) #107; modified from Murakami et al. (2012a, 2012b) #82; derived from Fordyce, (1994); Tanaka and Fordyce, (2014, 2015) #83; Tanaka et al. (2017) #84).
3. Inflections of premaxillae just anterior to, or in line with, anterior edge of supraorbital processes of frontals: absent (0); present (1) (Murakami et al. (2012a, 2012b) #83; modified from Geisler and Sanders, (2003) #108; Geisler et al., (2011) #108, (2012) #108; Tanaka and Fordyce, (2014, 2015) #84; Tanaka et al. (2017) #85).
4. Premaxillary cleft: absent (0); present, posterior part of ascending processes of premaxillae bearing a distinct cleft, originating at posterior edge of premaxillae and continuing anteriorly, dividing each premaxilla into two (1); present, with shallow cleft (2) (Geisler and Sanders, (2003) #109; Geisler et al., (2011) #109, (2012) #109; Murakami et al. (2012a, 2012b) #84; Tanaka and Fordyce, (2014, 2015) #85; Tanaka et al. (2017) #86).
5. Posterior end of nasal process of premaxillae (ordered): In addition to facing at least partially dorsally, face anterolaterally (0); face anteriorly (1); face anteromedially (2) (modified from Moore, (1968); Lambert, et al, (2017) #113).
6. Straight line distance between posterior-most point of right premaxilla along opening of bony external nares and the right nasal (ordered): Right premaxilla and nasal contact each other or are separated by a narrow gap that is < 15% of the maximum width of the external bony nares (0); intermediate separation, distance is between 17 and 50% of maximum nares width (1); wide, distance is > 60% of maximum nares width (2) (Lambert, et al, (2017) #307).
7. Orientation of the medial portion maxilla that is situated on either side of the vertex (ordered): Faces mainly laterally (0); faces mainly dorsolaterally (1); faces mainly dorsally (2) (Lambert, et al, (2017) #308).
8. Deep wedge of supraoccipital and/or interparietal between frontals on vertex: Absent (0); present (1) (Lambert, et al, (2017) #309).
9. Lateral margin of posterolateral sulcus: Low (0); high and as a result posterolateral sulcus is deeply entrenched (1) (Lambert, et al, (2017) #310).
10. Nasal bones: two (0); one or zero (1) (Heyning, (1989) #9, (1997) #41; Murakami et al. (2012a, 2012b) #85; modified from Messenger and McGuire, (1998) #1431; Geisler and Sanders, (2003) #113; Geisler et al., (2011) #113, (2012) #113; derived from Kuzmin, (1976); Tanaka and Fordyce, (2014, 2015) #86; Tanaka et al. (2017) #87).
11. Dorsoventral thickness of anterior edge of nasal (ordered): Very thin, nasal thickness < 82% of the anterior nasal width (0); thick, nasal thickness between 100% and 173% of the nasal width (1); very thick, > 200% the nasal width (2) (Lambert, et al, (2017) #116) Measurement taken approximately five millimeters from anterior edge.
12. Nasal (ordered): Elongate anteroposterior plate or blocky (0); anteroposteriorly compressed into a nearly vertical plate with fossa on ventrolateral surface for posterior nasal sac (caudal sac of Cranford et al., 1996) (1); fossa well excavated with boss delimiting dorsal edge (2) (modified from Muizon, (1988); Messenger & McGuire, (1998); Lambert, et al, (2017) #118).
13. Combined width of posterior edge of nasals (ordered): Wide, width > 150% the maximum width of external bony nares (0); subequal to external nares, width between 85% and 135% the nares width (1); narrow, width between 79% and 50% the nares width (2); very narrow, width between 44% and 39% the nares width (3); extremely narrow, width < 31% the nares width (4) (Lambert, et al, (2017) #121) The combined width can only be measured where the nasals meet on the midline. In some taxa there is a more posterior point where the width is wider, but the nasals, in such cases, are separated medially by the frontals.
14. Height of posterior portions of nasals relative to lateral edge of maxilla (ordered): Approximately equal to height of the base of rostrum; nasal height between 92% and 139% the rostral height (0); elevated above rostrum, height of nasals between 156% and 203% the rostral height (1); very elevated, height of nasals between 229% and 282% the rostral height (2); extremely elevated, height of nasals between 354% and 420% the rostral height (3); nasals tower above facial part of skull, height of nasals > 548% the rostral height (4) (modified from Heyning, (1989); Lambert, et al, (2017) #124).
15. Longest side of nasal faces (ordered): Dorsally (0); mainly anterodorsal (1); mainly anteriorly (2) (Lambert, et al, (2017) #312).
16. Premaxillae between anteromedial sulci emanating from premaxillary foramen (prenarial triangle): Convex transversely and rise up towards midline (0); rise up towards midline but concave transversely (1); flat or concave, may slightly rise up towards midline but not noticeably so (2) (Lambert, et al, (2017) #317).
17. Canal and/or foramen near posterior end of nasal process of premaxilla near suture with maxilla (ordered): Absent (0); present, foramen occurs between the maxilla laterally and the premaxilla medially, lateral or just anterior to the bony external nares, a sulcus continues posteriorly on the maxilla (1); same as sate “1” but the maxilla medial to the sulcus from the foramen is curled over to turn part of the sulcus into a canal (2) (Lambert, et al, (2017) #318).
18. Posteromedial sulcus of the premaxilla: Present (0); absent or barely visible (1) (Lambert, et al, (2017) #319).
19. Premaxillae anterior to nasal openings: Are flat or concave anteroposteriorly, form a premaxillary sac fossa (spiracular plate) (0); convex anteroposteriorly (1) (Lambert, et al, (2017) #320).
20. Medial half of premaxillary sac fossa (ordered). —Distinctly more excavated than lateral half and surface faces dorsomedially (0); either at same level as lateral half or fairly flat so that it primarily faces dorsally, extreme medial margin may be slightly upturned (1); ascends to form a prominent, longitudinal ridge (2) (Lambert, et al, (2017) #324).
21. Nasals: lower than frontals (0); nearly same height as frontals (1); clearly higher than frontals (2) (Muizon, (1988); Messenger and McGuire, (1998) #1434; Geisler and Sanders, (2003); #124; Geisler et al., (2011) #124, (2012) #124; Murakami et al. (2012a) #86; Tanaka and Fordyce, (2014, 2015) #87; Tanaka et al. (2017) #88).
22. Nasal protuberance: absent (0); present (1) (Muizon, (1988); Messenger and McGuire, (1998) #1433; Fajardo-Mellor et al., (2006) #7; Lambert, (2008) #8; Murakami et al. (2012a, 2012b) #87; Tanaka and Fordyce, (2014, 2015) #88; Tanaka et al. (2017) #89).
23. Both nasals: straight anterior edges in one transverse plane (0); with point on midline and gap on each side between premaxilla and nasal (1); concave posteriorly on midline and gap on each side between premaxilla and nasal (2); concave posteriorly on midline (3) (Murakami et al. (2012a, 2012b) #88; modified from Geisler and Sanders, (2003) #116; Geisler et al., (2011) #116, (2012) #116; derived from Moore, (1968); Tanaka and Fordyce, (2014, 2015) #89; Tanaka et al. (2017) #90).
24. Nasals: fossae on nasals absent (0); smooth-surfaced fossa on anterior to anterolateral surface (1) (Messenger and McGuire, (1998) #1437; Murakami et al. (2012a, 2012b) #89; Tanaka and Fordyce, (2014, 2015) #90; Tanaka et al. (2017) #91).
25. Transverse width of either of nasals as percent maximum length of nasals: very narrow, <20% (0); narrow, 21–69% (1); wide, >70% (2) (Murakami et al. (2012a) #90; modified from Muizon, (1988); Messenger and McGuire, (1998) #1432; Geisler and Sanders, (2003); #119; Geisler et al., (2011) #119, (2012) #119; Tanaka and Fordyce, (2014, 2015) #91; Tanaka et al. (2017) #92).
26. Nasals: medial portions roughly in same horizontal plane as, or higher than, lateral portions (0); medial portions depressed, forming a median trough immediately posterior to nasal openings (1) (Muizon, (1988, 1991); Geisler and Sanders, (2003); #118; Geisler et al., (2011) #118, (2012) #118; Murakami et al. (2012a, 2012b) #91; Tanaka and Fordyce, (2014, 2015) #92; Tanaka et al. (2017) #93).
27. Lateral edges of nasals: not overhanging or covering maxillae or premaxillae (0); overhanging or partly covering maxillae or premaxillae (1) (Murakami et al. (2012a, 2012b) #92; Tanaka and Fordyce, (2014, 2015) #93; Tanaka et al. (2017) #94).
28. Anterolateral corner of nasal (ordered): Lacks a distinct process (0); bears a thin process that extends anteriorly but is not inflated (1); has a distinct, inflated process that tapers in width anteriorly and ventrally, in lateral view, the angle between the dorsal and anterior face of the process is rounded over (2); same as state “2” but in anterior view, there is a crease that is oriented dorsomedially and separates the distinct process from the rest of the nasal (3) (Lambert, et al, (2017) #321) In taxa with nasals that roof the nasal cavity, the crease would be on the ventral surface of the nasal, although so far it has only been observed in taxa with highly reduced nasals.
29. Posterior part of suture between nasals. —Fairly flat (0); marked by a deep cleft (1) (Muizon, (1988); Lambert, et al, (2017) #323).
30. Nasal-frontal suture: approximately straight transversely (0); anterior wedge (narial process) between frontal posterior ends of nasals (1); W or reversed U suture line (2) (Murakami et al. (2012a, 2012b) #93; modified from Muizon, (1988); Geisler and Sanders, (2003) #121; Geisler et al., (2011) #121, (2012) #121; Tanaka and Fordyce, (2014, 2015) #94; Tanaka et al. (2017) #95).
31. Dorsal exposure of frontals: Fairly flat with separation between right and left frontal obscure (0); frontals are nodular with distinct separating sulcus on midline (1) (Fordyce, (1994); Lambert, et al, (2017) #127).
32. Frontals posterior to nasals and between premaxillae: wider than maximum transverse width across nasals (0); same as transverse width of nasals (1); narrower than transverse width of nasals, maxillae expanded medially posterior to nasals (2) (Geisler and Sanders, (2003)#125; Geisler et al., (2011) #125, (2012) #125; Murakami et al. (2012a, 2012b) #94; modified from Messenger and McGuire, (1998) #1457; Tanaka and Fordyce, (2014, 2015) #95; Tanaka et al. (2017) #96).
33. Frontal boss on vertex: absent (0); present (1) (Muizon, (1984, 1988); Messenger and McGuire, (1998) #1461; Fajardo-Mellor et al., (2006) #12; Murakami et al. (2012a, 2012b) #95; modified from Lambert, (2008) #9; Tanaka and Fordyce, (2014, 2015) #96; Tanaka et al. (2017) #97).
34. Vertex: absent (0); present (1); highly developed (2) (Murakami et al. (2012a) #96; Muizon, (1991); Messenger and McGuire, (1998) #1404; Lambert, (2005) #7; Tanaka and Fordyce, (2014, 2015) #97; Tanaka et al. (2017) #98).
35. Cranial vertex skewed asymmetrically to left side: absent (0); present (1) (Barnes, (1990); Bianucci, (2005) #7; Aguirre-Fernandez et al., (2009) #18; Murakami et al. (2012a, 2012b) #97; Tanaka and Fordyce, (2014, 2015) #98; Tanaka et al. (2017) #99).
36. Anterodorsal wall of braincase: formed by frontals (0); mostly formed by maxillae (1) (Geisler and Sanders, (2003) #127; Geisler et al., (2011) #127, (2012) #127; Murakami et al. (2012a, 2012b) #98; derived from Schulte, (1917); Miller, (1923); Tanaka and Fordyce, (2014, 2015) #99; Tanaka et al. (2017) #100).
37. Nuchal crest: higher than frontals and/or nasals (0); at same level as frontals and/or nasals (1); below frontals and/or nasals (2) (Murakami et al. (2012a, 2012b) #99; modified from Geisler and Sanders, (2003) #128; derived from Moore, (1968); Tanaka and Fordyce, (2014, 2015) #100; Tanaka et al. (2017) #101).

**Temporal Fossae, Zygomatic Arch, and Occipitals**

1. Temporal crest: Dorsal surface adjacent to crest is nearly horizontal, crest appears to be directed laterally (0); dorsal surface is concave and surface of temporal fossa below crest faces almost entirely laterally, crest appears to be oriented dorsolaterally (1) (Muizon, (1988); Lambert, et al, (2017) #131).
2. Frontal/parietal suture in lateral view: Vertical or slightly angled posteroventrally (0); dorsal portion of suture pointed and extended far anterior so that the anteriormost point of parietal is anterior to the posteriormost point of premaxilla (1) (Miller, (1923); Lambert, et al, (2017) #134).
3. Cross-section through intertemporal region, including parietals (ordered): Ovoid cross-section with sagittal crest (0); ovoid but sagittal crest absent (1); pinched ventrally and dorsal part expanded laterally, expanded part is rounded-over in crosssection (2); dorsal part is greatly expanded, overhangs more ventral portions, and lateral edge of dorsal surface is a sharp ridge (3) (Lambert, et al, (2017) #137).
4. Length of intertemporal region, ventral view of skull roof with basicranium removed (ordered): Intertemporal region absent or short, canal in frontals that contained olfactory stem and bulbs is < 10% of the length from posterior edge of dorsal nasal sinuses to posterior edge of skull (0); long, canal is between 18% and 35% of that length (1); very long, canal > 44% of that length (2) (Lambert, et al, (2017) #138).
5. Dorsoventral thickness of intertemporal region (ordered): Thin, thickness is < 25% the maximum height of skull, as measured from intercondyloid notch to dorsal-most point of the supraoccipital (0); thick, thickness between 30% and 43% of the skull height (1); very thick, thickness > 54% of the skull height (2) (Lambert, et al, (2017) #139).
6. Anteriormost point of the supraoccipital, in dorsal view (ordered): In transverse line with space between posterior edge of skull and anterior edge of the floor of squamosal fossa (0); in line with space between anterior edge of the floor of squamosal fossa and the anterior tip of zygomatic process of squamosal (1); in line with gap between anterior edge of zygomatic process of squamosal and the anteriormost point along posterior edge of the supraorbital process of frontal (2); in line with supraorbital process of frontal (3); in line with or anterior to anterior edge of supraorbital process of frontal; anterior edge of supraorbital process is taken at its medialmost point (4) (modified from Miller, (1923); Lambert, et al, (2017) #140).
7. Temporal fossa shape in lateral view: height lower than anteroposterior length (0); higher (1); nearly equilateral square (2); lower and its posterior end is rounded (3) (Tanaka and Fordyce, (2014, 2015) #281; Tanaka et al. (2017) #102).
8. Temporal fossa: not roofed over by lateral expansion of maxillae (0); roofed over by lateral expansion of maxillae (1) (Muizon, (1988); Heyning, (1989) #22, (1997) #54; Arnold and Heinsohn,(1996) #39; Messenger and McGuire, (1998) #1453; Murakami et al. (2012a, 2012b) #100; Tanaka and Fordyce, (2014, 2015) #101; Tanaka et al. (2017) #103).
9. Roof of temporal fossa formed by: frontals (0); frontals, but with large opening through maxillae and/or premaxillae exposing margins of window formed by a frontal ring (1) (Geisler and Sanders, (2003) #132; Geisler et al., (2011) #132, (2012) #132; Murakami et al. (2012a, 2012b) #101; Tanaka and Fordyce, (2014, 2015) #102; Tanaka et al. (2017) #104).
10. Position and orientation of origin for temporal muscle on supraorbital process of frontal: origin laying on posterior face of supraorbital process and directed roughly posteriorly (0); origin lying on posteroventral face of supraorbital process and directed roughly ventrally (1) (Fordyce, (1994) #8; Lambert, (2005) #23; Murakami et al. (2012a, 2012b) #102; Tanaka and Fordyce, (2014, 2015) #103; Tanaka et al. (2017) #105).
11. Parietal dorsally: not fused to frontal or supraoccipital (0); completely fused to, and indistinguishable from, frontal or supraoccipital (1) (Murakami et al. (2012a, 2012b) #103; Tanaka and Fordyce, (2014, 2015) #104; Tanaka et al. (2017) #106).
12. Parietals in dorsal view: contacting each other on the midline or separated by interparietal (0); in skull roof but visible only as small triangular areas at edges of intertemporal constriction, with supraoccipital overlapping and obscuring median portions (1); completely absent in skull roof (2); visible only as triangular areas, dorsolateral to supraoccipital, with non-overlapping supraoccipital separated from and contacting parietals along irregular suture (3) (Geisler and Sanders, (2003) #134; Geisler et al., (2011) #134, (2012) #134; Murakami et al. (2012a, 2012b) #104; derived from Whitmore and Sanders, (1977); Barnes, (1990); modified from Lambert, (2005) #15; Tanaka and Fordyce, (2014, 2015) #105; Tanaka et al. (2017) #107).
13. Interparietal: present (0); absent or fused and therefore not distinguishable from parietals and frontals (1) (Geisler and Sanders, (2003) #135; Geisler et al., (2011) #135, (2012) #135; Murakami et al. (2012a) #105; Tanaka and Fordyce, (2014, 2015) #106; Tanaka et al. (2017) #108).
14. Sagittal crest for temporal muscle: present (0); absent (1) (Murakami et al. (2012a, 2012b) #106; modified from Geisler and Sanders, (2003) #136; Geisler et al., (2011) #136, (2012) #136; Tanaka and Fordyce, (2014, 2015) #107; Tanaka et al. (2017) #109).
15. Alisphenoid: broadly exposed laterally in temporal fossa (0); lateral surface broadly overlapped by parietal, with a narrow strip visible or invisible on ventral edge of temporal fossa in lateral view (1) (Geisler and Sanders, (2003) #141; Geisler et al., (2011) #141, (2012) #141; Murakami et al. (2012a) #107; Tanaka and Fordyce, (2014, 2015) #108; Tanaka et al. (2017) #110).
16. Anterior zygomatic process end of squmosal in lateral view: taipered (0); squared (1) (Tanaka and Fordyce, (2014, 2015) #282; Tanaka et al. (2017) #111).
17. Zygomatic process of squamosal: directed anterolaterally (0); directed anteriorly (1) (Sanders and Barnes, 2002; Geisler and Sanders, (2003) #142; Geisler et al., (2011) #142, (2012) #142; Murakami et al. (2012a, 2012b) #108; Tanaka and Fordyce, (2014, 2015) #109; Tanaka et al. (2017) #112).
18. Zygomatic process of squamosal in lateral view: part of dorsal face visible (0); entire dorsal surface of squamosal visible (1) (Murakami et al. (2012a, 2012b) #109; Tanaka and Fordyce, (2014, 2015) #110; Tanaka et al. (2017) #113).
19. Dorsal edge of zygomatic process, skull in lateral view: Gently convex dorsally (0); near anterior end there is a distinct dorsal flange or process, flange usually articulates with frontal (1); concave dorsally (2) (Lambert, et al, (2017) #144).
20. Depth of squamosal fossa (ordered): Absent or very shallow, depth of fossa < 52% the horizontal distance from the supramastoid crest to the point above deepest part of squamosal fossa (0); shallow, depth between 55% and 91% that distance (1); deep, depth between 98% and 168% that distance (2); very deep, depth greater than 80% that distance (3) (derived from Barnes, (1985b); Lambert, et al, (2017) #147). The depth of the fossa is measured relative to the level of the supramastoid crest (i.e., dorsal edge of the zygomatic process) which forms the lateral border of the fossa.
21. Longitudinal profile of floor of squamosal fossa: Highly sigmoidal, concave posteriorly in region of a secondary squamosal fossa (sensu Sanders and Barnes, (2002)) but convex anteriorly (0); slightly sigmoidal, posterior part concave but does not form a discrete pit (1); flat (2); convex (3) (Lambert, et al, (2017) #148).
22. Floor of squamosal fossa: Same dorsoventral thickness anteriorly and posteriorly (0); thickens posteriorly (1) (Lambert, et al, (2017) #149).
23. Ventral edge of zygomatic process of squamosal in lateral view (ordered): Concave ventrally (0); straight (1); convex ventrally (2) (Lambert, et al, (2017) #151).
24. Emargination of posterior edge of zygomatic process by neck muscle fossa, skull in lateral view: absent, posterior edge forming nearly right angle with dorsal edge of zygomatic process of squamosal (0); shallow emargination (1); deep emargination (2) (Geisler and Sanders, (2003) #144; Geisler et al., (2011) #144, (2012) #144; Murakami et al. (2012a, 2012b) #110; Tanaka and Fordyce, (2014, 2015) #111; Tanaka et al. (2017) #114).
25. Width of squamosal lateral to exoccipital as percent greatest width of exoccipitals, skull in posterior view: exposed portion of squamosal narrow, <15% (0); moderate, 16–35% (1) (modified from Geisler and Sanders, (2003) #145; Geisler et al., (2011) #145, (2012) #145; Murakami et al. (2012a, 2012b) #111; Tanaka and Fordyce, (2014, 2015) #112; Tanaka et al. (2017) #115).
26. Postglenoid process of squamosal: not reduced (0); greatly reduced (1) (Murakami et al. (2012a, 2012b) #113; Tanaka and Fordyce, (2014, 2015) #114; Tanaka et al. (2017) #116).
27. Postglenoid process in lateral view: tapering ventrally (0); squared off ventrally (1); same as state 1 except very wide anteroposterior diameter of process (2) (Geisler and Sanders, (2003) #151; Lambert, (2005) #24; Geisler et al., (2011) #151, (2012) #151; Murakami et al. (2012a, 2012b) #114; derived from Muizon, (1991); Tanaka and Fordyce, (2014, 2015) #115; Tanaka et al. (2017) #117).
28. Relative ventral projections of postglenoid and post-tympanic processes of squamosal: postglenoid process more ventral or at same level as post-tympanic process (0); apex of postglenoid process dorsally higher than post-tympanic process (1) (Lambert, (2005) #25; Murakami et al. (2012a, 2012b) #115; Tanaka and Fordyce, (2014, 2015) #116; Tanaka et al. (2017) #118).
29. Nuchal crest in dorsoposterior view: semicircular, pointed anteriorly (0); rectangular or weakly convex anteriorly or posteriorly (1); convex posteriorly and/or midpoint convex triangular and pointed anteriorly (2); prominently convex anteriorly (3); strongly convex posteriorly (4) (Murakami et al. (2012a, 2012b) #116; modified from Geisler and Sanders, (2003); #152; Geisler et al., (2011) #152, (2012) #152; derived from Barnes, (1985b); Tanaka and Fordyce, (2014, 2015) #117; Tanaka et al. (2017) #119).
30. Deep sagittal sulcus on middle of occiput: Absent (0); present (1) (Lambert, et al, (2017) #311).
31. Occipital (ordered): Concave (0); flat or nearly so (1); convex (2); bulbous with inflated braincase (3) (Barnes, (1990); Lambert, et al, (2017) #313).
32. Dorsal condyloid fossa: absent (0); present, situated anterodorsal to dorsal edge of condyle (1); present and forming deep pit (2) (Geisler and Sanders, (2003) #156; Geisler et al., (2011) #156, (2012) #156; Murakami et al. (2012a, 2012b) #118; derived from Sanders and Barnes, (2002); Tanaka and Fordyce, (2014, 2015) #119; Tanaka et al. (2017) #121).
33. Lambdoidal crests of supraoccipital: Horizontal and directed laterally, overhanging temporal fossae (0); directed dorsolaterally, not or only slightly overhanging temporal fossae (1); very low and not directed either way (2) (Lambert, et al, (2017) #154) This crest is in a region where it can also be called the temporal crest or the nuchal crest (see Mead & Fordyce, (2009)).

**Anterior Basicranium**

1. Palatine/Maxilla suture: In ventral view, suture between both palatines and both maxillae is straight transversely or bowed anteriorly (0); maxillae have posterior processes that separate palatines anteriorly, suture around midline is V-shaped and points posteriorly (1) (Lambert, et al, (2017) #21).
2. Palatine (ordered): Ventral surface flat or convex (0); bears fossa for anterior end of pterygoid sinus (1); fossa well developed, divides palatine into medial and lateral laminae (2) (Muizon, (1988, 1991); Lambert, et al, (2017) #161).
3. Palatine in nasal passage: thin, forming posterior part of nasal passage (0); thick, forming part of anterior wall of nasal cavities (1); palatine does not join anterior wall of nasal passage (2) (Murakami et al. (2012a, 2012b) #119; modified from Geisler and Sanders, (2003); #158; Geisler et al., (2011) #158, (2012) #158; derived from Miller, (1923); Tanaka and Fordyce, (2014, 2015) #120; Tanaka et al. (2017) #122).
4. Palatine exposure: exposed ventrally (0); partially covered by pterygoid, which divides it into medial and lateral exposures (1); ventral surfaces completely covered by pterygoids (2) (Muizon, (1987); Arnold and Heinsohn,(1996) #15; Messenger and McGuire, (1998) #1440; Geisler and Sanders, (2003) #159; Lambert, (2005) #27; Geisler et al., (2011) #159, (2012) #159; Murakami et al. (2012a, 2012b) #120; derived from Miller, (1923); Tanaka and Fordyce, (2014, 2015) #121; Tanaka et al. (2017) #123).
5. Lateral lamina of palatine: absent (0); present (1) (Muizon, (1984, 1988) (1991); Arnold and Heinsohn,(1996) #16; Messenger and McGuire, (1998) #1443; Murakami et al. (2012a, 2012b) #121; Tanaka and Fordyce, (2014, 2015) #122; Tanaka et al. (2017) #124).
6. Lateral lamina of palatine relationship with maxilla: free from or sutured to maxilla (0); fused to maxilla (1) (Muizon, (1988); Messenger and McGuire, (1998) #1439; Geisler and Sanders, (2003) #161; Geisler et al., (2011) #161, (2012) #161; Murakami et al. (2012a, 2012b) #122; Tanaka and Fordyce, (2014, 2015) #123; Tanaka et al. (2017) #125).
7. Lateral lamina of palatine relationship with orbit: does not form bony bridge “over” (= ventral to) orbit (0); does form bony bridge “over” (= ventral to) orbit (1) (Muizon, (1984); Messenger and McGuire, (1998) #1444; Murakami et al. (2012a, 2012b) #123; Tanaka and Fordyce, (2014, 2015) #124; Tanaka et al. (2017) #126).
8. Pterygoids in anteroventral view: separated from each other by posteroventrally elongated palatines and/or vomer (0); contacting entire length of hamular process (1); contacting each other partially (2) (Murakami et al. (2012a, 2012b) #124; modified from Arnold and Heinsohn,(1996) #5; Messenger and McGuire, (1998) #1445; Fajardo-Mellor et al., (2006) #9; derived from Flower, (1884); Barnes, (1985a); Marsh et al., (1989); Tanaka and Fordyce, (2014, 2015) #125; Tanaka et al. (2017) #127).
9. Medial pterygoid-palatine suture in ventral view: angled anteromedially (0); nearly transverse (1); angled anterolaterally (2); angled anteroposteriorly (3) (Murakami et al. (2012a, 2012b) #125; modified from Geisler and Sanders, (2003) #162; Geisler et al., (2011) #162, (2012) #162; Tanaka and Fordyce, (2014, 2015) #126; Tanaka et al. (2017) #128).
10. Lateral lamina of pterygoid: absent (0); present and articulated with alisphenoid (1); partial, restricted to region lateral to hamular process (2) (Murakami et al. (2012a) #126; modified from Arnold and Heinsohn,(1996) #121; Messenger and McGuire, (1998) #1446; Geisler and Sanders, (2003) #164; Lambert, (2005) #32; Geisler et al., (2011) #164, (2012) #164; derived from Miller, (1923); Kellogg, (1936); Fraser and Purves, (1960); Tanaka and Fordyce, (2014, 2015) #127; Tanaka et al. (2017) #129).
11. Subtemporal crest: present (0); present but reduced, or absent (1) (modified from Geisler and Sanders, (2003) #165; Geisler et al., (2011) #165, (2012) #165; Murakami et al. (2012a, 2012b) #127; Tanaka and Fordyce, (2014, 2015) #128; Tanaka et al. (2017) #130).
12. Superior lamina of pterygoid: absent from sphenoidal region but present in orbital region (0); present and covers most of ventral exposure of alisphenoid (1); partially absent from orbital region (2); completely absent from orbital region (3) (Murakami et al. (2012a, 2012b) #128; modified from Arnold and Heinsohn,(1996) #16; Geisler and Sanders, (2003) #167; Geisler et al., (2011) #167, (2012) #167; derived from Miller (1923); Fraser and Purves, (1960)); Tanaka and Fordyce, (2014, 2015) #129; Tanaka et al. (2017) #131).
13. Pterygoids excavated anterior to choanae by the pterygoid sinuses, with distinct anterior fossa clearly limited forwards by rounded wall: absent (0); present (1) (Lambert, (2005) #28; Murakami et al. (2012a, 2012b) #129; Tanaka and Fordyce, (2014, 2015) #130; Tanaka et al. (2017) #132).
14. Depth of pterygoid sinus fossa in basicranium: shallow or partially excavated (0); deep, excavated dorsally to level of cranial foramen oval (1); deep, and extended dorsally into orbit (2) (modified from Fordyce, (1994) #6; Lambert, (2005) #30; Murakami et al. (2012a, 2012b) #130; Tanaka and Fordyce, (2014, 2015) #131; Tanaka et al. (2017) #133).
15. Anterior level of pterygoid sinus fossa: interrupted posterior to, or the level of, antorbital notch (0); extending beyond the level of the antorbital notch (1) (Lambert, (2005) #29; Murakami et al. (2012a, 2012b) #131; Tanaka and Fordyce, (2014, 2015) #132; Tanaka et al. (2017) #134).
16. Preorbital and postorbital fossae of pterygoid sinuses: widened apices of preorbital and postorbital fossae of pterygoid sinuses present but fossae not merged or fused (0); widened apices of preorbital and postorbital fossae of pterygoid sinuses merged or fused dorsal to path of optic nerve (1) (Murakami et al. (2012a, 2012b) #132; modified from Muizon, (1988); Arnold and Heinsohn,(1996) #19; Bianucci, (2005) #10; Aguirre-Fernandez et al., (2009) #19; Tanaka and Fordyce, (2014, 2015) #133; Tanaka et al. (2017) #135).
17. Fossa for preorbital lobe of pterygoid sinus in orbit: absent (0); present (1) (Fraser and Purves, (1960); Arnold and Heinsohn,(1996) #18; Murakami et al. (2012a, 2012b) #133; Tanaka and Fordyce, (2014, 2015) #134; Tanaka et al. (2017) #136).
18. Dorsal development of fossa for preorbital lobe of pterygoid sinus toward the frontal-maxilla suture: absent (0); present (1) (Muizon, (1984, 1988); Heyning, (1989) #37, (1997) #69; Messenger and McGuire, (1998) #1460; Arnold and Heinsohn,(1996) #20; Lambert, (2008) #13; Murakami et al. (2012a, 2012b) #134; modified from Fajardo-Mellor et al., (2006) #13; derived from Fraser and Purves, (1960); Tanaka and Fordyce, (2014, 2015) #135; Tanaka et al. (2017) #137).
19. Postorbital lobe of pterygoid sinus fossa: absent (0); present (1) (Arnold and Heinsohn,(1996) #18; Geisler and Sanders, (2003) #170; Geisler et al., (2011) #170, (2012) #170; Murakami et al. (2012a, 2012b) #135; derived from Fraser and Purves, (1960); Tanaka and Fordyce, (2014, 2015) #136; Tanaka et al. (2017) #138).
20. Anteroposteriorly elongated pterygoid sinus fossa, at level of orbit, bordered by mediolaterally compressed subtemporal crest of frontal: absent (0); present (1) (Murakami et al. (2012a, 2012b) #136; Tanaka and Fordyce, (2014, 2015) #137; Tanaka et al. (2017) #139).
21. Orbitosphenoid: not contacting lacrimal or lacrimojugal (0); contacting lacrimal or lacrimojugal (1) (Murakami et al. (2012a, 2012b) #137; Tanaka and Fordyce, (2014, 2015) #138; Tanaka et al. (2017) #140).
22. Ratio of length of hamular process of pterygoid to cranium length: <0.30 (0); 0.30–0.44 (1); 0.45–0.59 (2); >0.60 (3). The length of the hamular process of the pterygoid is measured from anterior edge of the pterygoid to posterior edge of the hamular process. The cranium length is measured from anterior edge of the antorbital process to posterior edge of occipital condyles. (Murakami et al. (2012a, 2012b) #138; modified from Heyning, (1989) #18, (1997) #50; Muizon, (1991); Messenger and McGuire, (1998) #1447; Lambert, (2005) #31; Tanaka and Fordyce, (2014, 2015) #139; Tanaka et al. (2017) #141).
23. Keel affecting ventral surfaces of hamular processes: absent (0); present (1) (Muizon, (1988); Messenger and McGuire, (1998) #1449; Bianucci, (2005) #14; Murakami et al. (2012a, 2012b) #139; modified from Fajardo-Mellor et al., (2006) #10; Tanaka and Fordyce, (2014, 2015) #140; Tanaka et al. (2017) #142).
24. Exposure of medial lamina of pterygoid hamuli in lateral view: complete or broad exposure due to extreme reduction of lateral lamina of pterygoid hamuli (0); no exposure due to a posterior extension of lateral lamina extending posterior to medial lamina (1); medial lamina of pterygoid hamuli exposing lateral lamina through ovoid window in lateral view (2) (Muizon, (1988); Fajardo-Mellor et al., (2006) #11; Murakami et al. (2012a, 2012b) #140; derived from Noble and Fraser, (1971); Tanaka and Fordyce, (2014, 2015) #141; Tanaka et al. (2017) #143).
25. Superior lamina of pterygoid (ordered): Present and covers most of ventral exposure of alisphenoid (0); absent from the sphenoidal region but present in orbital region (1); partially absent from orbital region (2); completely absent from orbital region (3) (Miller, (1923); Fraser & Purves, (1960); Lambert, et al, (2017) #168).
26. Posterior part of pterygoid sinus fossa, region immediately anterior to exit for mandibular branch of trigeminal nerve (ordered): One single fossa (0); split into a smaller and shallower posterior fossa and a much larger anteriorly extended deeper fossa by a low ridge (1); same as state “1” except divided by a high ridge (2) (Lambert, et al, (2017) #169).
27. Hamular process of the pterygoid: Splint like (0); solid, long and subconical (1); hollow and excavated by pterygoid sinus, lateral side highly concave, may or may not have lateral and inferior laminae (2); form thin horizontal plates (3); absent (4) (modified from Fraser & Purves, (1960); Lambert, et al, (2017) #173).
28. Hamular processes of pterygoids: Rounded over in ventral view (0); bear anteroposterior keels (1) (Muizon, (1988); Lambert, et al, (2017) #174).
29. Posteriormost point of hamular process of pterygoid, or medial part of pterygoid if the hamular process is absent (ordered): In transverse line with the middle of orbit (0); in line with postorbital process (1); in line with anterior edge of zygomatic process of squamosal (2); in line with middle of zygomatic process (3); in line with the postglenoid process (4) (Lambert, et al, (2017) #175).
30. Shape of restricted area between postorbital ridge of frontal and subtemporal crest from ventral view: anteroposteriorly long elliptical (0); wide fan-shape (1); narrow fan-shape (2), rhombus (3) (Tanaka and Fordyce, (2014, 2015) #280; Tanaka et al. (2017) #144).

**Posterior Basicranium**

1. Fossa for pterygoid sinus on alisphenoid posterior to groove for mandibular branch of trigeminal nerve (ordered): Absent, bone is flat or not ossified because of enlarged internal foramen ovale (0); shallow fossa (1); deep subcircular fossa (2) (modified from Fordyce, (1994); Lambert, et al, (2017) #176).
2. Lateral edge of middle sinus: Smooth (0); deckle-edged (1) (derived from Fraser & Purves, (1960); Lambert, et al, (2017) #180).
3. Ventral part of squamosal posterior to postmeatic process (ordered): Large area of laminated bone, appears externally as multiple sutures (0); small area of laminated bone restricted to ventrolateral edge of squamosal (1); without laminated bone (2) (Kasuya, (1973); Lambert, et al, (2017) #183).
4. Falciform process of squamosal: plate-like with anteroposteriorly wide base (0); rod-like with narrow base (1); poorly developed or absent (2) (Geisler and Sanders, (2003) #176; Geisler et al., (2011) #176, (2012) #176; Murakami et al. (2012a, 2012b) #141; modified from Lambert, (2005) #36; Tanaka and Fordyce, (2014, 2015) #142; Tanaka et al. (2017) #145).
5. Falciform process of squamosal: medial surface not sutured to lateral lamina of pterygoid (0); medial surface sutured to lateral lamina of pterygoid (1) (Murakami et al. (2012a, 2012b) #142; modified from Geisler and Sanders, (2003) #177; Geisler et al., (2011) #177, (2012) #177; Tanaka and Fordyce, (2014, 2015) #143; Tanaka et al. (2017) #146).
6. Tympanosquamosal recess: absent, with anterior transverse ridge present (0); anterior transverse ridge absent and middle sinus inferred to be present without a large tympanosquamosal recess (1); present and enlarged, forming triangular fossa medial and anteromedial to postglenoid process (2); very large, forming large fossa bordering entire medial edge of glenoid fossa (3) (Geisler and Sanders, (2003) #178; Geisler et al., (2011) #178, (2012) #178; Murakami et al. (2012a, 2012b) #143; modified from Lambert, (2005) #35; derived from Fraser and Purves, (1960), and Fordyce, (2002); Tanaka and Fordyce, (2014, 2015) #144; Tanaka et al. (2017) #147).
7. Bifurcation of tympanosquamosal recess: absent, almost undeveloped, elliptic (0); present, with a clear expansion anteriorly, invasion of mandibular fossa medially, and a depression (expansion) at the postglenoid process posteriorly (1) (Murakami et al. (2012a, 2012b) #144; modified from Muizon, (1988); Bianucci, (2005) #11; Aguirre-Fernandez et al., (2009) #20; Tanaka and Fordyce, (2014, 2015) #145; Tanaka et al. (2017) #148).
8. Fossa for the basisphenoidal sinus: absent (0); present (1) (Fraser and Purves, (1960); Mead and Fordyce, (2009); Murakami et al. (2012a, 2012b) #145; Tanaka and Fordyce, (2014, 2015) #146; Tanaka et al. (2017) #149).
9. Position of more-distal part of alisphenoid-squamosal suture, with skull in ventral view: anterior to external opening of foramen oval or a homologous groove (0); courses along groove for mandibular branch of trigeminal nerve, or just posterior to it (1); just medial to anterior edge of floor of squamosal fossa, foramen ovale, and/or groove situated entirely on alisphenoid (2) (Geisler and Sanders, (2003) #180; Geisler et al., (2011) #180, (2012) #180; Murakami et al. (2012a, 2012b) #146; Tanaka and Fordyce, (2014, 2015) #147; Tanaka et al. (2017) #150).
10. Groove for mandibular branch of trigeminal nerve: lateral end of groove wrapping laterally around posterior end of pterygoid sinus fossa and opening primarily anteriorly (0); directed laterally and located entirely posterior to pterygoid sinus fossa (1) (Murakami et al. (2012a, 2012b) #147; modified from Geisler and Sanders, (2003) #181; Geisler et al., (2011) #181, (2012) #181; Tanaka and Fordyce, (2014, 2015) #148; Tanaka et al. (2017) #151).
11. Suprameatal pit of squamosal: absent (0); present but shallow, situated dorsolateral to spiny process of squamosal (1); forming deep dorsolateral excavation into squamosal (2) (Geisler and Sanders, (2003) #185; Geisler et al., (2011) #185, (2012) #185; Murakami et al. (2012a, 2012b) #149; Tanaka and Fordyce, (2014, 2015) #149; Tanaka et al. (2017) #152).
12. Foramen spinosum: absent (0); present, located in anteromedial corner of anterior part of periotic fossa near or on squamosal-parietal suture (1) (Muizon, (1994); Geisler and Sanders, (2003)#186; Geisler et al., (2011) #186, (2012) #186; Murakami et al. (2012a, 2012b) #150; Tanaka and Fordyce, (2014, 2015) #150; Tanaka et al. (2017) #153).
13. Cranial hiatus (ordered): Absent, periotic contacts basioccipital medially or partially separated by narrow fissure (0); present, wide space between basioccipital and both periotic and squamosal (1); present but constricted, a medial projection of parietal partially divides fenestra (2); absent, parietal contacts basisphenoid and/or basioccipital dividing fenestra in two, an anterior foramen ovale and a large posterior opening (3); absent and posterior opening in state “2” is subdivided into several smaller foramina for nerves and vessels (4) (modified from Heyning, (1989) and Luo & Gingerich, (1999); Lambert, et al, (2017) #184).
14. Periotic fossa: Bowl-shaped (0); has transverse ridge that divides it into anterior and posterior portions (1) (Lambert, et al, (2017) #185) Cannot be scored for taxa that lack a periotic fossa.
15. Posterior portion of periotic fossa of squamosal: fossa absent (0); fossa present but shallow (1); highly compressed fossa forming narrow slit or small blind foramen (2); posteromedial portion contains large deep fossa (3) (Geisler and Sanders, (2003) #187; Geisler et al., (2011) #187, (2012) #187; Murakami et al. (2012a, 2012b) #149 and #151; Tanaka and Fordyce, (2014, 2015) #151; Tanaka et al. (2017) #154).
16. Length of zygomatic process of squamosal as percent of greatest width of maxilla at postorbital process: >31% (0); ≤30% (1) (Murakami et al. (2012a, 2012b) #152; modified from Heyning, (1989) #33, 35, #65, 67; Geisler and Sanders, (2003) #188; Geisler et al., (2011) #188, (2012) #188; Tanaka and Fordyce, (2014, 2015) #152; Tanaka et al. (2017) #155).
17. External auditory meatus: wide (0); narrow (1) (Fordyce, (1994) #10; Geisler and Sanders, (2003) #189, (2012) #189; Lambert, (2005) #26; Geisler et al., (2011) #189; Murakami et al. (2012a, 2012b) #153; Tanaka et al. (2017) #156).
18. Vomer: posterior edge terminating on or at anterior edge of basisphenoid (0); terminating on basioccipital, covering basioccipital-basisphenoid suture ventrally (1) (Barnes, (1984b); Geisler and Sanders, (2003) #190; Geisler et al., (2011) #190, (2012) #190; Murakami et al. (2012a, 2012b) #154; Tanaka and Fordyce, (2014, 2015) #153; Tanaka et al. (2017) #157).
19. Rectus capitus anticus muscle fossa: absent or poorly developed (0); present with well-defined anterior edge (1) (Geisler and Sanders, (2003) #192; Geisler et al., (2011) #192, (2012) #192; Murakami et al. (2012a, 2012b) #155; Tanaka and Fordyce, (2014, 2015) #155; Tanaka et al. (2017) #158).
20. Posteroventral-most point of basioccipital crest: rounded over (0); forming closely appressed separate flange, with narrow crease separating exoccipital dorsally from rest of basioccipital crest (1); projecting distinct flange posteriorly (2); distinct but separated by pronounced notch, interrupting basioccipital crest (3) (Geisler and Sanders, (2003) #193; Geisler et al., (2011) #193, (2012) #193; Murakami et al. (2012a, 2012b) #156; Tanaka and Fordyce, (2014, 2015) #156; Tanaka et al. (2017) #159).
21. Angle formed by basioccipital crests in ventral view: parallel with no angle formed (0); ca. 15–40° (1); ca. 42–68° (2); ca. 70–90° (3); >100° (4) (Murakami et al. (2012a, 2012b) #157; modified from Geisler and Sanders, (2003) #194; Geisler et al., (2011) #194, (2012) #194; Tanaka and Fordyce, (2014, 2015) #157; Tanaka et al. (2017) #160).
22. Basioccipital width compared with maximum width of skull in ventral view: narrow, less than 50% (0), wider, larger than 51% (1) (Tanaka and Fordyce, (2014, 2015) #283; Tanaka et al. (2017) #161).
23. Hypoglossal foramen: separated from jugular foramen, or jugular notch, by thick bone (0); separated by very thin bone or absent, in latter case hypoglossal foramen becoming confluent with jugular foramen (1) (Geisler and Sanders, (2003) #195; Geisler et al., (2011) #195, (2012) #195; Murakami et al. (2012a, 2012b) #158; Tanaka and Fordyce, (2014, 2015) #158; Tanaka et al. (2017) #162).
24. Jugular notch, gap between paroccipital process and basioccipital crest: open notch, width of opening and depth of notch about equal (0); narrow and almost slit-like, depth much greater than width of opening (1) (Geisler and Sanders, (2003) #196; Geisler et al., (2011) #196, (2012) #196; Murakami et al. (2012a, 2012b) #159; Tanaka and Fordyce, (2014, 2015) #159; Tanaka and Fordyce, (2014, 2015) #159; Tanaka et al. (2017) #163).
25. Paroccipital process, skull in ventral view: posterior edge located well anterior to the posterior edge of condyle (0); posterior edge in transverse line with posterior edge of condyle (1) (Geisler and Sanders, (2003) #197; Geisler et al., (2011) #197, (2012) #197; Murakami et al. (2012a, 2012b) #160; Tanaka and Fordyce, (2014, 2015) #160; Tanaka et al. (2017) #164).
26. Fossa for posterior sinus in exoccipital: absent or slightly concave (0); moderately concave (1); forming deep sack-like structure (2) (Murakami et al. (2012a, 2012b) #161; modified from Muizon, (1991); Lambert, (2005) #38; Tanaka and Fordyce, (2014, 2015) #161; Tanaka et al. (2017) #165).
27. Occipital condyles; on pedicle (0); lacking pedicle, unified with occipital (1) (Tanaka and Fordyce, (2014, 2015) #284; Tanaka et al. (2017) #166).

**Malleus**

1. Tuberculum of malleus: unreduced (0); highly reduced, almost indistinguishable from articular head (1) (Muizon, (1985); Messenger and McGuire, (1998) #1499; Geisler and Sanders, (2003) #198; Geisler et al., (2011) #198, (2012) #198; Murakami et al. (2012a, 2012b) #162; modified from Lambert, (2005) #69. derived from Doran, (1878); Tanaka and Fordyce, (2014, 2015) #162; Tanaka et al. (2017) #167).
2. Processus muscularis of malleus: shorter than manubrium of malleus (0); sub-equal or longer than manubrium (1) (Murakami et al. (2012a, 2012b) #163; modified from Muizon, (1985; 1988); Messenger and McGuire, (1998) #1550; Geisler and Sanders, (2003) #199; Lambert, (2005) #70; Geisler et al., (2011) #199, (2012) #199; Tanaka and Fordyce, (2014, 2015) #163; Tanaka et al. (2017) #168).

**Periotic**

1. Length of anterior process of periotic as percent length of pars cochlearis: short, <59% (0); long, >60% (1) (Murakami et al. (2012a, 2012b) #164; modified from Muizon, (1988); Heyning, (1989) #5; Messenger and McGuire, (1998) #1489; Geisler and Luo, (1996) #1; Luo and Marsh, (1996) #24; Geisler and Sanders, (2003) #203; Lambert, (2005) #39; Geisler et al., (2011) #203, (2012) #203; derived from Kellogg, (1936); Yamada, (1953); Kasuya, (1973); Tanaka and Fordyce, (2014, 2015) #164; Tanaka et al. (2017) #169).
2. Apex of anterior process of periotic in dorsal view: pointed (0); dorsal edge of anterior process showing highly rounded or oblique edge due to its reduction with or without pointed apex (1); thickened by prominent dorsal tubercle giving apex rectangular section in plane of body of periotic (2) (Murakami et al. (2012a, 2012b) #165; modified from Fordyce, (1994) #53; Lambert, (2005) #40; Tanaka and Fordyce, (2014, 2015) #165; Tanaka et al. (2017) #170).
3. Lateral groove or depression affecting profile of periotic as viewed dorsally: no obvious vertical groove dorsal to hiatus epitympanicus (0); groove present with overall profile of periotic becoming slightly to markedly sigmoidal in dorsal view (1) (Fordyce, (1994) #35; Murakami et al. (2012a, 2012b) #166; Tanaka and Fordyce, (2014, 2015) #166; Tanaka et al. (2017) #171).
4. Profile of anterior process of periotic ventrally deflected in lateral view: no, has crudely rectangular profile (0); smoothly deflected (1); abruptly deflected (2) (Fordyce (1994) #25; (Tanaka and Fordyce, (2014, 2015) #288; Tanaka et al. (2017) #172).
5. Anteroposterior ridge on dorsal side: undeveloped (0); developed on anterior process and body of periotic, associated with development of depression adjacent to groove for tensor tympani (1) (Fordyce, (1994) #55; Murakami et al. (2012a, 2012b) #167; Tanaka and Fordyce, (2014, 2015) #167; Tanaka et al. (2017) #173).
6. Anterior process in lateral view: Anterior edge of anterior process squared-off (0); comes to a blunt apex (1); comes to a slender point (2) (modified from Muizon, (1988); Lambert, et al, (2017) #202).
7. Apex of anterior process of the periotic: At same level or dorsal to ventral edge of pars cochlearis (0); well ventral to ventral edge of the pars cochlearis, process appears to be ventrally deflected (1) (modified from Fordyce, (1994); Lambert, et al, (2017) #203).
8. Anterior process in lateral view: Ventral edge convex ventrally or nearly flat (0); ventral edge clearly concave (1) (Muizon, (1988); Lambert, et al, (2017) #205). The fovea epitubaria, which articulates with the accessory ossicle of the tympanic, is greatly expanded anteriorly in state “1”.
9. Anteroexternal sulcus: Absent (0); present on lateral surface of anterior process of periotic, oriented primarily anteroposteriorly but bowed ventrally (1) (modified from Fordyce, (1994); Lambert, et al, (2017) #206).
10. Sulcus for capsuloparietal emissary vein: Present, forms a dorsoventral groove on lateral side of anterior process immediately anterior to lateral tuberosity (0); absent (1) (derived from Geisler & Luo, (1998); Lambert, et al, (2017) #207).
11. Shape of cross section through anterior process at midlength (ordered): Highly elliptical, transverse diameter is < 36% the dorsoventral diameter (0); ovoid, transverse diameter is between 51% and 78% of the dorsoventral diameter (1); approximately circular, transverse diameter between 85% and 134% of the dorsoventral diameter (2); bulbous, transverse diameter > 141% of the dorsoventral diameter (3) (modified from Fordyce, (1994) and Luo & Marsh, (1996); Lambert, et al, (2017) #209).
12. Contact of anterior process of periotic with portion of tympanic bulla anterior to accessory ossicle (ordered): Absent (0); present but no clear fossa for articulation on periotic (1); anterior bullar facet present but shallow with poorly defined medial edge (2); present with well-defined medial and lateral edges (3) (Fordyce, (1994); Lambert, et al, (2017) #210). Cannot be scored for taxa in which the bulla is completely fused to the anterior process of the periotic or in some taxa with very short anterior processes.
13. Articulation of anterior process of periotic to outer lip of tympanic bulla: contact of ventral surface of anterior process of periotic with outer lip of tympanic bulla (0); contact with thickened rim of outer lip of tympanic bulla and additionally with accessory ossicle (1); contact only with accessory ossicle (2) (Luo and Marsh, (1996) #7; Lambert, (2005) #46; Murakami et al. (2012a, 2012b) #168; Tanaka and Fordyce, (2014, 2015) #168; Tanaka et al. (2017) #174).
14. Parabullary sulcus: absent (0); strongly curved, C-shape (1); weakly curved (2); strongly curved, V-shape (3) (modified from Fordyce, (1994) #56 Anteroexternal sulcus; Tanaka and Fordyce, (2014, 2015) #169; Tanaka et al. (2017) #175).
15. Parabullary ridge of periotic: absent (0); present (1); present with a fossa between anterior process and parabullary ridge (2) (modified from Murakami et al. (2012a, 2012b) #171; Bianucci, (2005) #15; Tanaka and Fordyce, (2014, 2015) #170; Tanaka et al. (2017) #176).
16. Articulation of anterior process with squamosal: extensive, most of lateral side contacting squamosal (0); large centrally-oriented ovoid region contacting squamosal, free around edges (1); small area of contact with squamosal (2); contact absent, articulation via ligaments (3) (Geisler and Sanders, (2003) #207; Geisler et al., (2011) #207, (2012) #207; Murakami et al. (2012a, 2012b) #172; modified from Heyning, (1997) #32; Messenger and McGuire, (1998) #1490; derived from Heyning, (1989); Tanaka and Fordyce, (2014, 2015) #171; Tanaka et al. (2017) #177).
17. Anterior bullar facet: present (0); absent (1) (Muizon, (1984, 1988, 1991); Messenger and McGuire, (1998) #1496; Lambert, (2005) #42; Murakami et al. (2012a, 2012b) #173; modified from Fordyce, (1994) #4; derived from Kellogg, (1936); Tanaka and Fordyce, (2014, 2015) #172; Tanaka et al. (2017) #178).
18. Anterior incisure: deep, pocket-like fossa with anterior groove (0); anterior groove only (1) (Geisler and Luo, (1996) #7; Luo and Marsh, (1996) #15; Geisler and Sanders, (2003) #217; Geisler et al., (2011) #217, (2012) #217; Murakami et al. (2012a, 2012b) #174; Tanaka and Fordyce, (2014, 2015) #173; Tanaka et al. (2017) #179).
19. Lateral tuberosity (ordered): Absent (0); present, forms a bulbous prominence lateral to fossa for malleus (1); present and elongate, forms a lateral process that articulates dorsally with squamosal (2) (Muizon, (1991); Luo & Marsh, (1996); Geisler & Luo, (1996); Lambert, et al, (2017) #212).
20. Fossa incudis (ordered): Poorly defined or cannot be differentiated from rest of epitympanic recess (0); forms a clear circular fossa (1); circular fossa present on a short pedestal, the incudal process (2) (Luo & Marsh, (1996); Lambert, et al, (2017) #214).
21. Lateral side of periotic (ordered): Entire side of periotic contains pitted and rugose bone (0); all but anterior process is rugose (1); lateral side of posterior process of periotic is pitted and rugose, remaining portions are smooth (2); entire side of periotic is smooth (3) (Lambert, et al, (2017) #217).
22. Fenestra rotunda: oval to subrounded (0); shaped like teardrop with fissure directed toward aperture for cochlear aqueduct (1) (Fordyce, (1994) #22; Geisler and Sanders, (2003) #222; Lambert, (2005) #49; Geisler et al., (2011) #222, (2012) #222; Murakami et al. (2012a, 2012b) #175; Tanaka and Fordyce, (2014, 2015) #174; Tanaka et al. (2017) #180).
23. Dorsal surface of periotic in lateral view: convex dorsally (0); pyramidal process convex dorsally (1); nearly flat (2) (Murakami et al. (2012a, 2012b) #176; modified from Luo and Marsh, (1996) #18; Tanaka and Fordyce, (2014, 2015) #175; Tanaka et al. (2017) #181).
24. Relative position of dorsal depth of stapedial muscle fossa and fenestra rotunda: ventral to, or in line with, dorsal edge of fenestra rotunda (0); well dorsal to fenestra rotunda (1) (Geisler and Sanders, (2003) #223; Geisler et al., (2011) #223, (2012) #223; Murakami et al. (2012a, 2012b) #177; Tanaka and Fordyce, (2014, 2015) #176; Tanaka et al. (2017) #182).
25. Posterodorsal edge of stapedial muscle fossa: absent, rounded lip (0); present (1) (Geisler and Luo, (1996) #14; Geisler and Sanders, (2003) #217; Geisler et al., (2011) #217, (2012) #217; Murakami et al. (2012a, 2012b) #178; Tanaka and Fordyce, (2014, 2015) #177; Tanaka et al. (2017) #183).
26. Stylomastoid fossa (ordered): Absent (0); present, situated on posterior face of pars cochlearis posterodorsal to stapedial muscle fossa (1); enlarged dorsally and medially, covers much of posterior face of pars cochlearis (2); enlarged posterolaterally onto posterior process of the periotic (3) (Geisler & Luo, (1996); Lambert, et al, (2017) #225).
27. Caudal tympanic process of periotic in posteromedial view: Well separated from crista parotica, no division between stapedial muscle fossa and stylomastoid foramen (0); narrow separation or contact, clear separation of stapedial muscle fossa and stylomastoid foramen (1) (Lambert, et al, (2017) #227).
28. Facial nerve sulcus: Long sulcus on posterior process of periotic or the compound periotic/tympanic posterior process of most mysticetes (0); short, no sulcus posterior to stylomastoid notch (1) (Luo & Marsh, (1996); Geisler & Luo, (1996); Lambert, et al, (2017) #245).
29. Caudal tympanic process of periotic: low, its ventral and posterior edges drawing smooth curve (0); Elevated, its ventral and posterior edges forming a right angle in medial view (1) (Geisler and Sanders, (2003) #225; Geisler et al., (2011) #225, (2012) #225; Murakami et al. (2012a, 2012b) #179; Tanaka and Fordyce, (2014, 2015) #178; Tanaka et al. (2017) #184).
30. Distance between aperture of cochlear aqueduct and fenestra rotunda (ordered): No distance, both apertures are confluent (0); narrow, distance < 89% of the distance between fenestra ovalis and fenestra rotunda (1); wide, distance between 96% and 122% of the space between fenestrae ovalis and rotunda (2); very wide, distance > 146% (3) (modified from Geisler & Luo, (1996); Lambert, et al, (2017) #229).
31. Distance between aperture of vestibular aqueduct and fenestra rotunda (ordered): Very narrow, distance < 112% of the distance between fenestra ovalis and fenestra rotunda (0); narrow, distance between 121% and 185% (1); wide, distance between 192% and 211% (2); very wide, distance > 222% the distance between fenestra ovalis and fenestra rotunda (3) (Lambert, et al, (2017) #230).
32. Foramen singulare (ordered): In common recess with the spiral cribiform tract, transverse crest separating the foramen singulare from proximal opening of facial nerve canal is well developed (0); in common recess with spiral cribiform tract, transverse crest separating it from proximal opening of facial nerve canal is low, and proximal opening of facial nerve canal within internal acoustic meatus (1); separated by partitions of equal height from spiral cribiform tract and proximal opening of facial nerve canal (2); in common recess with proximal opening of facial nerve canal (3) (modified from Luo & Marsh, (1996); Geisler & Luo, (1996); Lambert, et al, (2017) #237).
33. Proximal opening of facial nerve canal (ordered): Anterior to spiral cribriform tract (0); slightly anterior, posterior edge of proximal opening of facial nerve canal is lateral to center of spiral cribiform tract (1); lateral to spiral cribriform tract (2) (Lambert, et al, (2017) #238).
34. Morphology of proximal opening of facial nerve canal: Continuous with an anterior fissure (0); oval-shaped (1); circular (2) (Luo & Marsh, (1996); Lambert, et al, (2017) #239).
35. Aperture for cochlear aqueduct: smaller than aperture for vestibular aqueduct (0); approximately same size as aperture for vestibular aqueduct (1); much larger than aperture for vestibular aqueduct, with narrow posterior edge (2) (Geisler and Sanders, (2003) #227; Geisler et al., (2011) #227, (2012) #227; Murakami et al. (2012a, 2012b) #181; modified from Muizon, (1987); Fordyce, (1994); Lambert, (2005) #52; Tanaka and Fordyce, (2014, 2015) #180; Tanaka et al. (2017) #186).
36. Excavation of tegmen tympani at base of anterior process: absent (0); present, with fossa on dorsolateral side of tegmen tympani (1) (Geisler and Sanders, (2003) #231; Geisler et al., (2011) #231, (2012) #231; Murakami et al. (2012a, 2012b) #182; Tanaka and Fordyce, (2014, 2015) #181; Tanaka et al. (2017) #187).
37. Fundus of internal acoustic meatus: funnel-like, smaller at blind end and wider near rim (0); tubular (1) (Luo and Marsh, (1996) #31; Geisler and Sanders, (2003) #234; Geisler et al., (2011) #234, (2012) #234; Murakami et al. (2012a, 2012b) #183; Tanaka and Fordyce, (2014, 2015) #182; Tanaka et al. (2017) #188).
38. Internal acoustic meatus: pyriform (0); circular (1) (Muizon, (1984); Messenger and McGuire, (1998) #1498; Bianucci, (2005) #21; Murakami et al. (2012a, 2012b) #184; Tanaka and Fordyce, (2014, 2015) #183; Tanaka et al. (2017) #189).
39. Lateral wall of internal acoustic meatus: high, with wedge-shaped area of elevated bone occurring between dorsal edge of tegmen tympani and internal acoustic meatus, the latter extending ventrally and increasing its depth (0); low, not protruding noticeably from fossa and surrounding bone (1) (Murakami et al. (2012a, 2012b) #185; modified from Geisler and Sanders, (2003) #235; Geisler et al., (2011) #235, (2012) #235; Tanaka and Fordyce, (2014, 2015) #184; Tanaka et al. (2017) #190).
40. Dorsal edge of tegmen tympani dorsolateral to internal acoustic meatus and anterior process (ordered): Present and high, dorsoventral height > 114% the width of pars cochlearis (0); present, height between 58% and 34% of promontorial width (1); low, height between 23% and 11% (2); forms a low ridge or is absent, height < 4% of the width of pars cochlearis (3) (derived from Fordyce, (1994); Lambert, et al, (2017) #233).
41. Dorsal edge of tegmen tympani lateral to aperture for vestibular aqueduct (ordered): Present and very high, dorsoventral height > 112% of the width of pars cochlearis (0); high, height between 95% and 50% that width (1); low, height between 12% and 4% (2); faint ridge (3); absent (4) (Lambert, et al, (2017) #234).
42. Cochlear aqueduct on periotic with a thin edge: no (0); yes (1) (Fordyce (1994) #28; Tanaka and Fordyce, (2014, 2015) #289; Tanaka et al. (2017) #191).
43. Thickness dorsoventral of pars cochlearis on periotic; thick (0) thin (1) (Gutstein et al (2014); Tanaka and Fordyce, (2014, 2015) #290; Tanaka et al. (2017) #192).
44. Profile of cochlear on periotic in dorsoventral; rounded (0), sub-rectangular (1), squared (2) (modified from Fordyce (1994) #61 and Bianucci et al (2013) #2; Tanaka and Fordyce, (2014, 2015) #291; Tanaka et al. (2017) #193).
45. Angle between anterior process of periotic and anterior edge of pars cochlearis (ordered): Obtuse, pars cochlearis appears transversely compressed (0); nearly 90 degrees, pars cochlearis looks rectangular or semicircular in ventral view (1); acute, pars cochlearis looks globular (2) (Lambert, et al, (2017) #219) Covers part of character 18 of Luo & Marsh (1996).
46. Pars cochlearis: Most convex part is on ventrolateral surface (0); most convex part is on medial surface. Area of greatest convexity begins anteromedial to fenestra rotunda and extends anterodorsally on the medial face. With periotic in dorsal view, there is a wide expanse of bone medial to internal acoustic meatus (1) (Lambert, et al, (2017) #221).
47. Aperture for vestibular aqueduct, in dorsal view: at transverse level of spiral cribriform tract (0); more lateral than spiral cribriform tract (1) (Lambert, (2005) #53; Murakami et al. (2012a, 2012b) #186; Tanaka and Fordyce, (2014, 2015) #185; Tanaka et al. (2017) #194).
48. Articular rim: absent (0); present but small, forming ridge anterolateral to articulation surface of posterior process of periotic and separated from it by sulcus (1); present, sigmoidal and laterally elongated with hook-like process (2) (Geisler and Sanders, (2003) #239; Geisler et al. (2012; 2011) #239; modified from Murakami et al. (2012a, 2012b) #187; modified from Muizon (1987); Messenger (1994); Messenger and McGuire (1998) #1494; Fordyce, (1994) #33; Lambert, (2005) #55; Tanaka and Fordyce, (2014, 2015) #186; Tanaka et al. (2017) #195).
49. Contact of periotic, not including anterior process, with skull (ordered): Distal end of posterior process of periotic, lateral surface of posterior process of the periotic, and entire dorsal edge of tegmen tympani (or homologous bone) contact squamosal and possibly parietal (0); same as state “0” except dorsal edge contacts from posterior end of posterior process of periotic to region just lateral to aperture of vestibular aqueduct (1); only dorsal and lateral sides of posterior process articulate with squamosal (2); periotic articulates with squamosal along hiatus epitympanicus and adjacent regions on the posterior process (3); periotic only articulates with skull via ligaments (4) (Lambert, et al, (2017) #241).
50. Posterior process of periotic in lateral view: ventrally bent (0); in same plane as body of periotic (1) (Bianucci, (2005) #19; Murakami et al. (2012a, 2012b) #189; modified from Arnold and Heinsohn,(1996) #28; Lambert, (2005) #54; Tanaka and Fordyce, (2014, 2015) #188; Tanaka et al. (2017) #197).
51. Angle between posterior process of periotic and long axis of pars cochlearis from dorsal or ventral views: >135° (0); ≤135° (1) (Murakami et al. (2012a, 2012b) #190; modified from Geisler and Sanders, (2003) #246; Lambert, (2005) #54; Geisler et al., (2011) #246, (2012) #246; derived from Kasuya, (1973); Barnes, (1990); Luo and Marsh, (1996); Tanaka and Fordyce, (2014, 2015) #189; Tanaka et al. (2017) #198).
52. Facet for bulla on posterior process of periotic, parallel-sided; no (0); yes (1) (modified from Fordyce, (1994) #63; Tanaka and Fordyce, (2014, 2015) #190; Tanaka et al. (2017) #199).
53. Ventral surface of posterior process of periotic, along a straight path perpendicular to its long axis: flat (0); concave (1); convex (2) (Murakami et al. (2012a, 2012b) #191; modified from Geisler and Sanders, (2003) #242; Geisler et al., (2011) #242, (2012) #242; Tanaka and Fordyce, (2014, 2015) #191; Tanaka et al. (2017) #200).
54. Posterior bullar facet of periotic: with many long deep grooves and low ridges (0); with some shallow grooves and/or low ridges (1); without grooves or ridges (2) (Bianucci, (2005) #20; Murakami et al. (2012a, 2012b) #192; Tanaka and Fordyce, (2014, 2015) #192; Tanaka et al. (2017) #201).
55. Length of posterior process of periotic as percent length of pars cochlearis: long, ≥85% (0); short, ≤84% (1) (Murakami et al. (2012a, 2012b) #193; modified from Barnes, (1990); Luo and Marsh, (1996) #24; Geisler and Sanders, (2003) #245; Geisler et al., (2011) #245, (2012) #245; Tanaka and Fordyce, (2014, 2015) #193; Tanaka et al. (2017) #202).
56. Mastoid exposure of posterior process of periotic on outside of skull: exposed externally (0); not exposed, enclosed by exoccipital and squamosal (1) (Geisler and Luo, (1996) #28; Luo and Marsh, (1996) #28; Geisler and Sanders, (2003) #249; Geisler et al., (2011) #249, (2012) #249; Murakami et al. (2012a, 2012b) #194; Tanaka and Fordyce, (2014, 2015) #194; Tanaka et al. (2017) #203).
57. Dorsal edge of posterior process, periotic in lateral or medial view: Straight or convex ventrally (0); concave ventrally, helps to form the neck of posterior process of periotic (1) (modified from Geisler & Luo, (1996); Lambert, et al, (2017) #248).
58. Posterior process of periotic: Robust (0); horizontal plate and very thin for most of its length (1) (Luo & Marsh, (1996); Lambert, et al, (2017) #249).

**Tympanic Bulla**

1. Anterior spine of tympanic bulla: absent (0); present but short (1); present and long (2) (Muizon, (1987); Fordyce, (1994) #45; Geisler and Sanders, (2003) #250; Lambert, (2005) #62; Geisler et al., (2011) #250, (2012) #250;Murakami et al. (2012a, 2012b) #195; modified from Messenger and McGuire, (1998) #1484; derived from Kasuya, (1973); Tanaka and Fordyce, (2014, 2015) #195; Tanaka et al. (2017) #204).
2. Anterolateral convexity of tympanic bulla with anterolateral notch: absent (0); present (1) (Muizon, (1987); Fordyce, (1994) #46; Lambert, (2005) #63; Murakami et al. (2012a, 2012b)#196; Tanaka and Fordyce, (2014, 2015) #196; Tanaka et al. (2017) #205).
3. Articulation of posterior process of tympanic bulla with squamosal: process contacting post-tympanic process of squamosal and posterior process of periotic (0); process contacting periotic only (1) (Muizon, (1984); Fordyce, (1994) #29; Arnold and Heinsohn,(1996) #34; Messenger and McGuire, (1998) #1481; Lambert, (2005) #56; Murakami et al. (2012a, 2012b) #197; derived Kasuya, (1973); Tanaka and Fordyce, (2014, 2015) #197; Tanaka et al. (2017) #206).
4. Width of tympanic bulla as percentage of its length along its long axis: wide, ≥65% (0); narrow and long, ≤64% (1) (Geisler and Sanders, (2003) #251; Bianucci, (2005) #23; Geisler et al., (2011) #251, (2012) #251; Murakami et al. (2012a, 2012b) #198; derived from Kasuya, (1973); Tanaka and Fordyce, (2014, 2015) #198; Tanaka et al. (2017) #207).
5. Accessory ossicle or homologous region on lip of bulla: not fused to anterior process of periotic (0); fused to anterior process of periotic (1) (Barnes, (1990); Fordyce, (1994); Luo and Marsh, (1996); Geisler and Sanders, (2003) #255; Geisler et al., (2011) #255, (2012) #255; Murakami et al. (2012a, 2012b) #199; Tanaka and Fordyce, (2014, 2015) #199; Tanaka et al. (2017) #208).
6. Accessory ossicle: Absent (0); present (1) (Luo & Marsh, (1996); Lambert, et al, (2017) #254).
7. Accessory ossicle: Small and oblong (0); large and subspherical (1) (Fordyce, (1994); Luo & Marsh, (1996); Lambert, et al, (2017) #255). Cannot be scored for taxa that lack an accessory ossicle.
8. Lateral furrow of tympanic bulla: shallow groove (0); absent (1); deep, well-defined groove (2) (Murakami et al. (2012a, 2012b) #200; modified from Muizon, (1984, 1988); Arnold and Heinsohn,(1996) #31; Messenger and McGuire, (1998) #1485; Fajardo-Mellor et al., (2006) #17; Lambert, (2008) #17; derived from Kasuya, (1973); Tanaka and Fordyce, (2014, 2015) #200; Tanaka et al. (2017) #209).
9. Sigmoid process (ordered): Forms a straight transverse plate that is directed perpendicular to long axis of bulla (0); forms a curved plate; proximal part is directed posterolaterally while the distal end curves to point laterally (1) (Kasuya, (1973); Lambert, et al, (2017) #259).
10. Sigmoid process: directed laterally to posterolaterally (0); directed anteriorly to anterolaterally (1) (Murakami et al. (2012a, 2012b) #201; modified from Messenger and McGuire, (1998) #1486; Lambert, (2005) #67, Kasuya, (1973); Tanaka and Fordyce, (2014, 2015) #201; Tanaka et al. (2017) #210).
11. Dorsomedial edge of sigmoid process: expanded anteriorly to appose lateral tuberosity of periotic (0); not articulating with squamosal or periotic (1) (Murakami et al. (2012a, 2012b) #202; modified from Geisler and Sanders, (2003) #260; Geisler et al., (2011) #260, (2012) #260; modified from Luo and Marsh, (1996) #10; Tanaka and Fordyce, (2014, 2015) #202; Tanaka et al. (2017) #211).
12. Ventral margin of tympanic bulla in lateral view: convex (0); concave (1) (Lambert, (2005) #66; Murakami et al. (2012a, 2012b) #203; Tanaka and Fordyce, (2014, 2015) #203; Tanaka et al. (2017) #212).
13. Elliptical foramen of tympanic bulla: present (0); absent or close (1) (Geisler and Sanders, (2003) #261; Geisler et al., (2011) #261, (2012) #261; Murakami et al. (2012a, 2012b) #204; derived from Kasuya, (1973); Tanaka and Fordyce, (2014, 2015) #204; Tanaka et al. (2017) #213).
14. Thickness of posterior process of tympanic bulla: Thick in region ventral to facet for posterior process of periotic (0); forms a thin lamina (1) (Lambert, et al, (2017) #265).
15. Distal end of posterior process of tympanic bulla (ordered): Thinner or approximately the same thickness as more proximal portions (0); distal end thicker but not hypertrophied (1); hypertrophied in size, forms large nodular mass (2) (Flower, (1872); Kasuya, (1973); Lambert, et al, (2017) #266).
16. Size of posterior process of tympanic bulla: equal to or greater than total length of tympanic bulla (0); much smaller than total length of tympanic bulla (1) (Muizon, (1984, 1991); Heyning, (1989) #23, 29, (1997) #55, 61; Messenger and McGuire, (1998) #1482; Murakami et al. (2012a, 2012b) #205; modified from Lambert, (2005) #57; derived from Yamada, (1953); Kasuya, (1973); Tanaka and Fordyce, (2014, 2015) #205; Tanaka et al. (2017) #214).
17. Surface of posterior process of tympanic bulla: spiny or irregular edges (0); cauliflower-like bony growth (1); rounded and pachyostotic (2) (Muizon, (1991); Messenger and McGuire, (1998) #1483; Murakami et al. (2012a, 2012b) #206; derived from Kasuya, (1973); Tanaka and Fordyce, (2014, 2015) #206; Tanaka et al. (2017) #215).
18. Median furrow: short extension on ventral face anterior to interprominental notch (0); anterolateral curvature of median groove to connect to long lateral furrow on outer lip (1); median groove reaching an anterior level beyond lateral furrow, and often slightly curved laterally (2); long and deep rectilinear median groove reaching at least to base of anterior tip of tympanic bulla (3) (Lambert, (2005) #64; Murakami et al. (2012a, 2012b) #207; Tanaka and Fordyce, (2014, 2015) #207; Tanaka et al. (2017) #216).
19. Median furrow on posterior side of bulla: divided by a transverse ridge originating from involucrum (0); transverse ridge absent (1) (Geisler and Sanders, (2003) #267; Geisler et al., (2011) #267, (2012) #267; Murakami et al. (2012a, 2012b) #208; Tanaka and Fordyce, (2014, 2015) #208; Tanaka et al. (2017) #217).
20. Involucrum: Bears prominent transverse groove on dorsal surface that divides involucrum into a thicker posterior part and thinner anterior part (0); groove absent (1) (Lambert, et al, (2017) #272).
21. Posterior edge of medial prominence of involucrum: approximately in line with posterior edge of lateral prominence (0); distinctly anterior to posterior edge of lateral prominence (1) (Muizon, (1987); Geisler and Sanders, (2003) #269; Geisler et al., (2011) #269, (2012) #269; Murakami et al. (2012a, 2012b) #209; derived from Kasuya, (1973); Tanaka and Fordyce, (2014, 2015) #209; Tanaka et al. (2017) #218).
22. Dorsal margin of involucrum of tympanic bulla: not excavated (0); excavated just anterior to posterior process (1); excavated at mid-part of involucrum (2) (Muizon, (1988); Messenger and McGuire, (1998) #1487; Murakami et al. (2012a, 2012b) #210, 211; modified from Lambert, (2005) #60; Geisler and Sanders, (2003) #271; Geisler et al., (2011) #271, (2012) #271; Tanaka and Fordyce, (2014, 2015) #210; Tanaka et al. (2017) #219).
23. Ridge on inside of bulla: present, as transverse ridge extending laterally from involucrum and partially dividing cavum tympani into anterior and posterior portions (0); absent (1) (Geisler and Sanders, (2003) #272; Geisler et al., (2011) #272, (2012) #272; Murakami et al. (2012a, 2012b) #212; Tanaka and Fordyce, (2014, 2015) #211; Tanaka et al. (2017) #220).
24. Posterior end of ventromedial keel: Forms a smooth curve around posterior part of involucrum (0); protrudes and points medially (1) (Lambert, et al, (2017) #253)
25. Ventromedial keel of tympanic bulla: present along entire length (0); terminating approximately at level of lateral furrow or mid-point of the tympanic bulla (1); poorly defined along entire length (2) (Geisler and Sanders, (2003) #273; Geisler et al., (2011) #273, (2012) #273; Murakami et al. (2012a, 2012b) #213; derived from Kasuya, (1973); Tanaka and Fordyce, (2014, 2015) #212; Tanaka et al. (2017) #221).
26. Posterior end of ventromedial keel: not protruding and directed medially (0); protruding and directed medially (1) (Geisler and Sanders, (2003) #275; Geisler et al., (2011) #275, (2012) #275; Murakami et al. (2012a, 2012b) #214; Tanaka and Fordyce, (2014, 2015) #213; Tanaka et al. (2017) #222).

**Hyals**

1. Basihyal and thyrohyal connection: unfused (0); fused (1) (Murakami et al. (2012a, 2012b) #215; modified from Bianucci, (2005) #25; Tanaka and Fordyce, (2014, 2015) #214; Tanaka et al. (2017) #223).
2. Basihyal and thyrohyal shape: arched (0); angled (1) (Murakami et al. (2012a, 2012b) #216; modified from Bianucci, (2005) #25; Tanaka and Fordyce, (2014, 2015) #215; Tanaka et al. (2017) #224).

**Vertebrae**

1. Length of skull relative to prelumbar vertebral column (ordered): Very short, condylobasal length of skull from 77% to 55% length of cervical plus thoracic portions of vertebral column (0); short, skull length from 100 to 83% length of cervical plus thoracic portions of vertebral column (1); long, 123% to 114% prelumbar column length (2); very long, > 135% prelumbar column length (3) (derived from Miller, (1923); Lambert, et al, (2017) #277).
2. Atlas (ordered): Ventral process larger than dorsal process (0); both processes are subequal (1); dorsal process larger (2) (modified from Muizon, (1987, 1988); Lambert, et al, (2017) #278).
3. Dorsal transverse process of atlas: developed dorsolaterally (0); fused with ventral transverse process, with height of process greater than width (1); absent or rudimentary obtuse angle (2) (Murakami et al. (2012a, 2012b) #217; modified from Muizon, (1988); Barnes, (1990); Tanaka and Fordyce, (2014, 2015) #216; Tanaka et al. (2017) #225).
4. Roof of neural canal of atlas: arched (0); convex (1); straight (2) (Murakami et al. (2012a, 2012b) #218; Tanaka and Fordyce, (2014, 2015) #217; Tanaka et al. (2017) #226).
5. Postzygapophysis of axis in anterior view: appearing as crest, elongated dorsolaterally (0); appearing as rudimentary crest (1); not appearing (2) (Murakami et al. (2012a, 2012b) #219; Tanaka and Fordyce, (2014, 2015) #218; Tanaka et al. (2017) #227).
6. Cervical vertebrae: unfused (0); atlas and axis fused (1); C1–C3 or C1–C4 fused (2); C1–C6 or C1–C7 fused (3); C2–C7 fused (4) (Murakami et al. (2012a, 2012b) #220; modified from Arnold and Heinsohn,(1996) #9; Messenger and McGuire, (1998) #1501; Geisler and Sanders, (2003) #278, 279; Fajardo-Mellor et al., (2006) #18; Lambert, (2008) #18; Geisler et al., (2011) #278, 279, (2012) #278, 279; derived from Allen, (1923); Miller, (1923); Fraser and Noble, (1971); De Smet, (1977); Rommel, (1990); Tanaka and Fordyce, (2014, 2015) #219; Tanaka et al. (2017) #228).
7. Length of cervicals (C1–C7) as percent of height of vertebral body plus neural canal of atlas: long, >150% (0); short, <150% (1) (Murakami et al. (2012a, 2012b) #221; Tanaka and Fordyce, (2014, 2015) #220; Tanaka et al. (2017) #229).
8. Number of thoracic vertebrae (ordered): 18 to 17 (0); 16 to 15 (1); 14 (2); 13 (3); 12 (4); 11 (5); 10 or less (6) (Lambert, et al, (2017) #281).
9. Number of thoracic vertebrae with capitular articulations (ordered): 11 (0); 10 (1); 9 (2); 8 (3); 7 (4); 6 (5); 4 to 5 (6); 3 or less (7) (Sanders and Barnes, (2002); Lambert, et al, (2017) #282).
10. Capitular articulation facets of posterior vertebrae: facets gradually shift downward on sequential vertebrae to fuse with tubercular facets (0); facets abruptly shift from a position on neural arch to a pedestal, originating from centrum on subsequent vertebra (1) (Geisler and Sanders, (2003) #282; Geisler et al., (2011) #282, (2012) #282; Murakami et al. (2012a, 2012b) #223; derived from Flower, (1868); Miller, (1923); Tanaka and Fordyce, (2014, 2015) #222; Tanaka et al. (2017) #230).
11. Lateral edge of transverse processes of lumbar vertebrae: Oriented anteroposteriorly (0); angled anteromedially 45° or more, relative to a parasagittal plane (1) (Muizon, (1988); Lambert, et al, (2017) #284).
12. Centrum of anterior lumbar vertebrae (ordered): Short, length < 63% the width (0); long, length between 79% and 136% the width (1); very long, length > 147% the width (2) (Muizon, (1988); Barnes, (1990); Lambert, et al, (2017) #287). The width is measured across the anterior face of the centrum.
13. Number of lumbar vertebrae (ordered): One (0); 3 (1); 4 (2); 6 (3); 7 to 8 (4); 9 to 10 (5); 11 to 12 (6); 13 to 16 (7); 16 to 19 (8); > 19 (9) (Lambert, et al, (2017) #288).
14. Transverse processes of lumbar vertebrae: extend parallel to anterior and posterior borders (0); triangular (1) (Muizon, (1984; 1985; 1988); Messenger and McGuire, (1998) #1502; Geisler and Sanders, (2003) #285; Geisler et al., (2011) #285, (2012) #285; Murakami et al. (2012a, 2012b) #224; Tanaka and Fordyce, (2014, 2015) #223; Tanaka et al. (2017) #231).
15. Transverse processes of lumbar vertebrae: oriented ventrolaterally (0); oriented laterally and horizontally (1) (Geisler and Sanders, (2003) #284; Geisler et al., (2011) #284, (2012) #284; Murakami et al. (2012a, 2012b) #225; derived from Sanders and Barnes, (2002); Tanaka and Fordyce, (2014, 2015) #224; Tanaka et al. (2017) #232).
16. Ratio of greatest breadth of transverse process to width of centrum at anterior face in lumbar vertebrae: some or all lumbar vertebrae >2.5 (0); no lumbar vertebrae >2.5 (1) (Murakami et al. (2012a, 2012b) #226; Tanaka and Fordyce, (2014, 2015) #225; Tanaka et al. (2017) #233).
17. Number of caudal vertebrae (ordered): 13 to 15 (0); 16 to 19 (1); 20 to 23 (2); 24 to 27 (3); 27 to 30 (4); 30 to 33 (5); 34 to 60 (6) (Lambert, et al, (2017) #289).

**Sternum and Sternal Ribs**

1. Sternum: consists of four or five parts (0); consists of two or three parts (1); consists of single bone (2) (Murakami et al. (2012a, 2012b) #229; modified from Geisler and Sanders, (2003) #290; Geisler et al., (2011) #290; derived from Yablokov, (1964); Van Valen, (1968); Tanaka and Fordyce, (2014, 2015) #228; Tanaka et al. (2017) #234).
2. Ventrolateral processes on manubrium of sternum: absent (0); present but small, occur ventral to articulation surface of first costal cartilage or rib (1) (Muizon, (1988); Messenger and McGuire, (1998) #1503; Geisler and Sanders, (2003) #289; Geisler et al., (2011) #289, (2012) #289; Murakami et al. (2012a, 2012b) #230; derived from Klima et al., (1980); Tanaka and Fordyce, (2014, 2015) #229; Tanaka et al. (2017) #235).
3. Sternal ribs: unossified or ossification of fewer than five pairs (0); ossification of five pairs or more (1) (Murakami et al. (2012a, 2012b) #231; derived from Flower, (1867); Tanaka and Fordyce, (2014, 2015) #230; Tanaka et al. (2017) #236).

**Scapula**

1. Anterodorsal part of scapula: rounded (0); rounded and anterior edge pointed (1); almost rectilinear (2) (Murakami et al. (2012a, 2012b) #232; Tanaka and Fordyce, (2014, 2015) #232; Tanaka et al. (2017) #237).
2. Ventral projection on anterior border of scapula: absent (0); present (1) (Fajardo-Mellor et al., (2006) #26; Murakami et al. (2012a, 2012b) #233; derived from Noble and Fraser, (1971); Tanaka and Fordyce, (2014, 2015) #233; Tanaka et al. (2017) #238).
3. Anterior slope on scapula between anterior angle and midpoint of glenoid fossa with anterior and posterior margin of glenoid fossa on a plane: shallow, <35° (0); steeper, >35° (1) (modified from Murakami et al. (2012a, 2012b) #234; modified from Bianucci, (2005) #31; Tanaka and Fordyce, (2014, 2015) #234; Tanaka et al. (2017) #239).
4. Posterior slope on scapula, between scapula and midpoint of glenoid fossa with anterior and posterior margin of glenoid fossa on a plane: shallow, <25° (0); steeper, >25° (1) (modified from Murakami et al. (2012a, 2012b) #235; modified from Bianucci, (2005) #32; Tanaka and Fordyce, (2014, 2015) #235; Tanaka et al. (2017) #240).
5. Crest between infraspinous fossa and teres fossa: weakly developed (0); strongly developed (1) (Murakami et al. (2012a, 2012b) #236; Tanaka and Fordyce, (2014, 2015) #236; Tanaka et al. (2017) #241).
6. Coracoid process of scapula: not expanded distally (0); expanded distally (1); notably reduced or absent (2) (Murakami et al. (2012a, 2012b) #237; modified from Muizon, (1987, 1994); Messenger and McGuire, (1998) #1504; Geisler and Sanders, (2003) #292; Lambert, (2005) #73; Bianucci, (2005) #33; Geisler et al., (2011) #292; derived from True, (1904); Tanaka and Fordyce, (2014, 2015) #237; Tanaka et al. (2017) #242).
7. Coracoid process of scapula, with glenoid fossa: directed horizontally (0); directed nearly anterodorsally (1); directed anteroventrally (2) (modified from Murakami et al. (2012a, 2012b) #238; modified Barnes, (1990); Tanaka and Fordyce, (2014, 2015) #238; Tanaka et al. (2017) #243).
8. Acromion of scapula: narrow and not expanded distally (0); expanded distally (1) (Murakami et al. (2012a, 2012b) #239; modified from Bianucci, (2005) #34; Tanaka and Fordyce, (2014, 2015) #239; Tanaka et al. (2017) #244).
9. Acromion of scapula, when glenoid fossa direct ventrally: directed horizontally (0); directed anterodorsally (1); directed anteroventrally (2) (Murakami et al. (2012a, 2012b) #240; modified from Barnes, (1990); Tanaka and Fordyce, (2014, 2015) #240; Tanaka et al. (2017) #245).
10. Supraspinous fossa of scapula: present (0); absent or nearly absent (1) (Muizon, (1987, 1994); Geisler and Sanders, (2003) #293; Lambert, (2005) #72; Geisler et al., (2012; 2011) #293; Murakami et al. (2012a, 2012b) #241; Tanaka and Fordyce, (2014, 2015) #241; Tanaka et al. (2017) #246).
11. Acromion process of scapula lies on anterior edge, with loss of supraspinous fossa: no (0); yes (1) (Muizon (1987); Fordyce (1994) #48; Tanaka and Fordyce, (2014, 2015) #292; Tanaka et al. (2017) #247).
12. Coracoid process of scapula: present (0); absent (1) (Muizon 1987; Fordyce 1994 #49; Tanaka and Fordyce, (2014, 2015) #293; Tanaka et al. (2017) #248).

**Forelimb (except scapula)**

1. Ratio of length of humerus to length of radius: long, >1.1 (0); short, <0.8 (1) (Murakami et al. (2012a, 2012b) #242; modified from Sanders and Barnes, (2002); Geisler and Sanders, (2003) #297; Geisler et al., (2012; 2011) #297; Tanaka and Fordyce, (2014, 2015) #242; Tanaka et al. (2017) #249).
2. Location of apex of deltopectoral tuberosity of humerus: within proximal 65% of humerus (0); within distal 35% of humerus (1) (Murakami et al. (2012a, 2012b) #243; modified from Muizon, (1988); Messenger and McGuire, (1998) #1506; Geisler and Sanders, (2003) #295; Bianucci, (2005) #35; Geisler et al., (2011) #295, (2012) #295; Tanaka and Fordyce, (2014, 2015) #243; Tanaka et al. (2017) #250).
3. Prominent deltoid crest on anterior edge of humerus: present, forms greatest anteroposterior diameter along shaft (0); forming a knob-like tuberosity (1); tuberosity or crest absent (2) (Geisler and Sanders, (2003) #294; Geisler et al., (2011) #294, (2012) #294; Murakami et al. (2012a, 2012b) #244; derived from Sanders and Barnes, (2002); Tanaka and Fordyce, (2014, 2015) #244; Tanaka et al. (2017) #251).
4. Radial and ulnar facets of humerus in lateral view: facets forming a semicircular articulation surface (0); facets forming an obtuse angle (1) (Barnes, Barnes (1990); Geisler and Sanders, (2003) #296; Geisler et al., (2011) #296, (2012) #296; Murakami et al. (2012a, 2012b) #245; Tanaka and Fordyce, (2014, 2015) #245; Tanaka et al. (2017) #252).
5. Olecranon process: present as a distinct process (0); present as a slightly raised proximal posterior edge (1); absent (2) (Messenger and McGuire, (1998) #1507; Geisler and Sanders, (2003) #296; Geisler et al., (2011) #284, (2012) #284; Murakami et al. (2012a, 2012b) #246; modified from Muizon, (1984); Barnes, (1990); Arnold and Heinsohn,(1996) #10; Fajardo-Mellor et al., (2006) #28; derived from Howell, (1927); Bianucci (2005) #37; Tanaka and Fordyce, (2014, 2015) #246; Tanaka et al. (2017) #253).
6. Delto-pectoral tuberosity or farthest anterior point of crest (ordered): Closer to proximal head of humerus (0); approximately centered, proximodistally, on shaft (1); closer to distal end of humerus (2) (Muizon, (1988); Lambert, et al, (2017) #296).
7. Manus: Pentadactyl (0); tetradactyl or fewer digits (1) (Yablokov, (1965); Lambert, et al, (2017) #300).

**Soft Tissues**

1. Spermaceti organ: absent (0); present (1) (Fordyce, (1994) #17; Messenger and McGuire, (1998) #1511; Geisler and Sanders, (2003) #97; Geisler et al., (2011) #97, (2012) #97; Murakami et al. (2012a, 2012b) #247; derived from Norris and Harvey, (1972); Cranford et al., (1996); Tanaka and Fordyce, (2014, 2015) #247; Tanaka et al. (2017) #254).
2. Museau de singe: absent (0); present (1) (Messenger and McGuire, (1998) #1512; Murakami et al. (2012a, 2012b) #248; derived from Norris, (1964); Cranford et al., (1996); Tanaka and Fordyce, (2014, 2015) #248; Tanaka et al. (2017) #255).
3. Lateral lips of nasal plug: present (0); absent (1) (Messenger and McGuire, (1998) #1523; Murakami et al. (2012a, 2012b) #249; Tanaka and Fordyce, (2014, 2015) #249; Tanaka et al. (2017) #256).
4. Proximal sac: single frontal sac (0); sac complex, with nasofrontal sacs and vestibule (1) (Heyning, (1989) #6, 11, 17, (1997) #33, 43, 49; Fordyce, (1994) #16; Messenger and McGuire, (1998) #1531, 1532; Lambert, (2005) #18; Murakami et al. (2012a); Murakami et al. (2012b) #250; Tanaka and Fordyce, (2014, 2015) #250; Tanaka et al. (2017) #257).
5. Posterior nasal sacs: absent (0); present (1) (Heyning, (1989) #41, (1997) #73; Arnold and Heinsohn, (1996) #6; Messenger and McGuire, (1998) #1534; Murakami et al. (2012a, 2012b) #251; Tanaka and Fordyce, (2014, 2015) #251; Tanaka et al. (2017) #258).
6. Posterior nasal sacs: single (0); divided (1) (Messenger and McGuire, (1998) #1535; Murakami et al. (2012a) #252; Tanaka and Fordyce, (2014, 2015) #252; Tanaka et al. (2017) #259).
7. Anterior section of nasofrontal sac: absent (0); present (1) (Messenger and McGuire, (1998) #1536; Murakami et al. (2012a, 2012b) #253; Tanaka and Fordyce, (2014, 2015) #253; Tanaka et al. (2017) #260).
8. Anterior part of nasofrontal sac: smooth (0); trabeculate (1) (Messenger and McGuire, (1998) #1537; Fajardo-Mellor et al., (2006) #35; Murakami et al. (2012a, 2012b) #254; derived from Heyning, (1989); Tanaka and Fordyce, (2014, 2015) #254; Tanaka et al. (2017) #261).
9. Vestibular sac: absent (0); present (1); hypertrophied (2) (Heyning, (1989) #28, (1997) #60; Fordyce, (1994) #31; Arnold and Heinsohn,(1996) #1, 3; Messenger and McGuire, (1998) #1541; Lambert, (2005) #17; Fajardo-Mellor et al., (2006) #39; Murakami et al. (2012a, 2012b) #255; Tanaka and Fordyce, (2014, 2015) #255; Tanaka et al. (2017) #262).
10. Floor of vestibular sac (nasal sac): not rigid (0); rigid (1) (Heyning, (1989) #38, (1997) #70; Arnold and Heinsohn,(1996) #2; Messenger and McGuire, (1998) #1543; Fajardo-Mellor et al., (2006) #38; Murakami et al. (2012a, 2012b) #256; Tanaka and Fordyce, (2014, 2015) #256; Tanaka et al. (2017) #263).
11. Vestibular sac (nasal sac): undivided (0); bilaterally divided (1) (Messenger and McGuire, (1998) #1544; Fajardo-Mellor et al., (2006) #36; Murakami et al. (2012a, 2012b) #257; derived from Heyning, (1989); Tanaka and Fordyce, (2014, 2015) #257; Tanaka et al. (2017) #264).
12. Right and left sides of vestibular sac (nasal sac): same size (0); right side larger than left (1) (Heyning, (1989) #30, (1997) #62; Messenger and McGuire, (1998) #1545; Murakami et al. (2012a, 2012b) #258; Tanaka and Fordyce, (2014, 2015) #258; Tanaka et al. (2017) #265).
13. Intrinsic muscle in vestibular sac (nasal sac): absent (0); present (1) (Messenger and McGuire, (1998) #1546; Fajardo-Mellor et al., (2006) #37; Murakami et al. (2012a, 2012b) #259; derived from Mead, (1975); Tanaka and Fordyce, (2014, 2015) #259; Tanaka et al. (2017) #266).
14. Floor of vestibular sac (nasal sac): smooth (0); wrinkled (1) (Heyning, (1997) #70; Arnold and Heinsohn,(1996) #2; Messenger and McGuire, (1998) #1543; Murakami et al. (2012a, 2012b) #260; Tanaka and Fordyce, (2014, 2015) #260; Tanaka et al. (2017) #267).
15. Diagonal membrane: absent (0); present (1) (Messenger and McGuire, (1998) #1550; Murakami et al. (2012a, 2012b) #261; Heyning, (1989); Tanaka and Fordyce, (2014, 2015) #261; Tanaka et al. (2017) #268).
16. Spiracular cavity: slit-like (0); rounded (1) (Messenger and McGuire, (1998) #1552; Murakami et al. (2012a, 2012b) #262; Tanaka and Fordyce, (2014, 2015) #262; Tanaka et al. (2017) #269).
17. Pars posteroexternus muscle: absent (0); present (1) (Messenger and McGuire, (1998) #1553; Murakami et al. (2012a, 2012b) #263; Tanaka and Fordyce, (2014, 2015) #263; Tanaka et al. (2017) #270).
18. Pars intermedius muscle: absent (0); present (1) (Messenger and McGuire, (1998) #1554; Murakami et al. (2012a, 2012b) #264; Tanaka and Fordyce, (2014, 2015) #264; Tanaka et al. (2017) #271).
19. Pars posterointerus muscle: absent (0); present (1) (Messenger and McGuire, (1998) #1556; Murakami et al. (2012a, 2012b) #265; Tanaka and Fordyce, (2014, 2015) #265; Tanaka et al. (2017) #272).
20. Pars anterointerus muscle: one insertion (0); two insertions (1) (Messenger and McGuire, (1998) #1557; Murakami et al. (2012a, 2012b) #266; Tanaka and Fordyce, (2014, 2015) #266; Tanaka et al. (2017) #273).
21. Blowhole shape: longitudinal slit, may be slightly sigmoidal or angled (0); crescent, with apices pointed anteriorly (1); crescent, with apices pointed posteriorly, may be skewed (2); rectangular (3) (Murakami et al. (2012a, 2012b) #267; modified from Messenger and McGuire, (1998) #1525; Tanaka and Fordyce, (2014, 2015) #267; Tanaka et al. (2017) #274).
22. Soft tissues of nasal passages distal to bony external nares: separated for most of their length but confluent just proximal to blowhole (0); confluent (1) (Heyning, (1989); Fordyce, (1994) #20; Messenger and McGuire, (1998) #1529; Geisler and Sanders, (2003) #95; Lambert, (2005) #16; Geisler et al., (2011) #95, (2012) #95; Murakami et al. (2012a, 2012b) #268; Tanaka and Fordyce, (2014, 2015) #268; Tanaka et al. (2017) #275).
23. Distal sac: absent (0); present, situated immediately distal to museau de singe (1) (Murakami et al. (2012a, 2012b) #269; modified from Heyning, (1989) #12, (1997)#44; Fordyce, (1994) #14; Messenger and McGuire, (1998) #1533; Geisler and Sanders, (2003) #99, (2012) #99; Lambert, (2005) #19; Geisler et al., (2011) #99; Tanaka and Fordyce, (2014, 2015) #269; Tanaka et al. (2017) #276).
24. Blowhole ligament: absent (0); present (1) (Heyning, (1989) #15, (1997) #44; Fordyce, (1994) #13; Messenger and McGuire, (1998) #1527; Geisler and Sanders, (2003) #101; Lambert, (2005) #20; Geisler et al., (2011) #101, (2012) #101; Murakami et al. (2012a, 2012b) #270; Tanaka and Fordyce, (2014, 2015) #270; Tanaka et al. (2017) #277).
25. Blowhole ligament: not appressed against skull (0); appressed against skull (1) (Messenger and McGuire, (1998) #1528; Murakami et al. (2012a, 2012b) #271; Tanaka and Fordyce, (2014, 2015) #271; Tanaka et al. (2017) #278).
26. Cartilage on blowhole ligament: absent (0); present (1) (Messenger and McGuire, (1998) #1529; Murakami et al. (2012a, 2012b) #272; Tanaka and Fordyce, (2014, 2015) #272; Tanaka et al. (2017) #279).
27. Accessory sac: absent (0); present, forms small diverticulum of inferior vestibule and extends anterolaterally around the attachment of blowhole ligament to the premaxilla (1) (Messenger and McGuire, (1998) #1549; Geisler and Sanders, (2003) #106; Fajardo-Mellor et al., (2006) #40; Geisler et al., (2011) #106, (2012) #106; Murakami et al. (2012a, 2012b) #274; derived from Schenkkan, (1971); Mead, (1975); Heyning, (1989); Tanaka and Fordyce, (2014, 2015) #273; Tanaka et al. (2017) #280).
28. Esophageal forestomach: present (0); absent (1) (Geisler and Sanders, (2003) #300; Geisler et al., (2011) #300, (2012) #300; Murakami et al. (2012a, 2012b) #275; derived from Mead, (1989); Rice and Wolman, (1990); Tanaka and Fordyce, (2014, 2015) #274; Tanaka et al. (2017) #281).
29. External throat grooves: absent (0); one pair converged anteriorly (1); irregular in number and shape (2) (Murakami et al. (2012a) #276; modified from Messenger and McGuire, (1998) #1512, 1513; Geisler and Sanders, (2003) #301; Geisler et al., (2011) #301, (2012) #301; Tanaka and Fordyce, (2014, 2015) #276; Tanaka et al. (2017) #282).
30. Dorsal fin: present (0); dorsal hump (1); absent (2) (Murakami et al. (2012a) #277; modified from Messenger and McGuire, (1998) #1562; Geisler and Sanders, (2003) #304; Geisler and Sanders, 2003 #304, (2012) #304; derived from Leatherwood and Reeves, (1983); Jefferson and Newcomer (1993), Reeves et al. (2002); Tanaka and Fordyce, (2014, 2015) #277; Tanaka et al. (2017) #283).
31. Shape of flipper: fan shaped (0); rounded at tip (1); sharply pointed at tip (2); entire flipper rounded (3) (Murakami et al. (2012a) #278; modified from Fajardo-Mellor et al., (2006) #31; Lambert, (2008) #25; derived from Leatherwood and Reeves (1983), Brownell et al. (1987); Tanaka and Fordyce, (2014, 2015) #278; Tanaka et al. (2017) #284).

**References**

Aguirre-Fernández, G., Barnes, L.G., Aranda-Manteca, F.J., and Fernández-Rivera, J.R. 2009. Protoglobicephala mexicana, a new genus and species of Pliocene fossil dolphin (Cetacea; Odontoceti; Delphinidae) from the Gulf of California, Mexico. Boletin de la Sociedad Geologica Mexicana, 61(2):245–265.

Allen, G.M. 1923. The black finless porpoise, Meomeris. Bulletin of the Museum of Comparative Zoology, 65(7):233–256.

Arnold, P.W., and Heinsohn, G.E. 1996. Phylogenetic status of the Irrawaddy dolphin *Orcaella brevirostris* (Owen in Gray): a cladistic analysis. Memoirs of the Queensland Museum, 39(2):141–204.

Barnes, L.G. 1984a. Fossil odontocetes (Mammalia: Cetacea) from the Almejas Formation, Isla Cedros, Mexico. Paleobios, 42:1–46.

Barnes, L.G. 1984b. Whales, dolphins and porpoises; origin and evolution of the Cetacea. In Gingerich, P.D., and Badgle, C.E. (eds.), Mammals. Notes for a short course, University of Atennessee, Department of Geological Science.

Barnes, L.G. 1985a. Evolution, taxonomy and antitropical distributions of the porpoises (Phocoenidae, Mammalia). Marine Mammal Science, 1(2):149–165.

Barnes, L.G. 1985b. Fossil pontoporiid dolphins (Mammalia: Cetacea) from the Pacific coast of North America. Contributions to Science, Natural History Museum of Los Angeles County, 363:1–34.

Barnes, L.G. 1990. The fossil record and evolutionary relationships of the genus Tursiops, p. 3–26. In Leatherwood, S., and Reeves, R.R. (eds.), The bottlenose dolphin. Academic Press Inc, San Diego, New York.

Bianucci, G. 2005. *Arimidelphis sorbinii* a new small killer whale-like dolphin from the Pliocene of Marecchia River (Central eastern Italy) and a phylogenetic analysis of the Orcininae (Cetacea: Odontoceti). Rivista Italiana di Paleontologia e Stratigrafia, 111(2):329–344.

Bianucci, G., Lambert, O., Salas-Gismondi, R., Tejada, J., Pujos, F., Urbina, M., and Antoine, P.-O. 2013. A Miocene relative of the Ganges River dolphin (Odontoceti, Platanistidae) from the Amazonian Basin. Journal of Vertebrate Paleontology, 33(3):741–745.

Brownell, R.L., Findley, L.T., Vidal, O., Robles, A., and Silvia Manzanilla, N. 1987. External morphology and pigmentation of the vaquita, *Phocoena sinus* (Cetacea: Mammalia). Marine Mammal Science, 3(1):22–30.

Cranford, T.W., Amundin, M., and Norris, K.S. 1996. Functional morphology and homology in the odontocete nasal complex: implications for sound generation. Journal of Morphology, 228(3):223–285.

Curry, B.E. 1992. Facial anatomy and potential function of facial structures for sound production in the harbor porpoise (*Phocoena phocoena*) and Dall's porpoise (*Phocoenoides dalli*). Canadian Journal of Zoology, 70(11):2103–2114.

De Smet, W.M.A. 1977. The regions of the cetacean vertebral column volume 3, p. 59–80. In Harrison, R.J. (ed.), Functional anatomy of marine mammals. Academic Press, London.

Doran, A.H. 1878. Morphology of the mammalian ossicula auditus. Transactions of the Linnaean Society, Series 2, Zoology, 1(18):371–497.

Fajardo‐Mellor, L., Berta, A., Brownell, R.L., Boy, C.C., and Goodall, N.P. 2006. The phylogenetic relationships and biogeography of true porpoises (Mammalia: Phocoenidae) based on morphological data. Marine Mammal Science, 22(4):910–932.

Flower, W.H. 1867. Description of the skeleton of *Inia geoffrensis* and the skull of *Pontoporia blainvillii*, with remarks on the systematic position of these animals in the Order Cetacea. Transactions of the Zoological Society of London, 6(3):87–116.

Flower, W.H. 1868. On the osteology of the cachalot or sperm-whale (*Physeter macrocephalus*). Transactions of the Zoological Society of London, 6(6):309–372.

Flower, W.H. 1872. On the recent ziphioid whales, with a description of the skeleton of *Berardius arnouxi*. Transactions of the Zoological Society of London, 8(3):203–234.

Flower, W.H. 1884. On the characters and divisions of the Family Delphinidae. Proceedings of the Zoological Society of London, 1883:466–513.

Flower, W.H. 1885. List of the specimens of Cetacea in the Zoological Department of the British Museum. British Museum, London.

Fordyce, R.E. 1994. *Waipatia maerewhenua*, new genus and new species (Waipatiidae, new family), an archaic Late Oligocene dolphin (Cetacea: Odontoceti: Platanistoidea) from New Zealand. Proceedings of the San Diego Society of Natural History, 29:147–176.

Fordyce, R.E. 2002. *Simocetus rayi* (Odontoceti: Simocetidae, new family): A bizarre new archaic Oligocene dolphin from the eastern North Pacific. Smithsonian Contributions to Paleobiology, 93:185–222.

Fraser, F.C., and Purves, P.E. 1960. Hearing in cetaceans: evolution of the accessory air sacs and the structure of the outer and middle ear in recent cetaceans. Bulletin of the British Museum of Natural History (Zoology) 7:1–140.

Geisler, J.H., Godfrey, S.J., and Lambert, O. 2012. A new genus and species of late Miocene inioid (Cetacea, Odontoceti) from the Meherrin River, North Carolina, USA. Journal of Vertebrate Paleontology, 32(1):198–211.

Geisler, J.H., and Luo, Z. 1996. The petrosal and inner ear of *Herpetocetus* sp.(Mammalia: Cetacea) and their implications for the phylogeny and hearing of archaic mysticetes. Journal of Paleontology:1045–1066.

Geisler, J.H., McGowen, M.R., Yang, G., and Gatesy, J. 2011. A supermatrix analysis of genomic, morphological, and paleontological data from crown Cetacea. BMC Evolutionary Biology, 11:1–33.

Geisler, J.H., and Sanders, A.E. 2003. Morphological evidence for the phylogeny of Cetacea. Journal of Mammalian Evolution, 10(1/2):23–129.

Gutstein, C.S., Figueroa-Bravo, C.P., Pyenson, N.D., Yury-Yañez, R.E., Cozzuol, M.A., and Canals, M. 2014. High frequency echolocation, ear morphology, and the marine–freshwater transition: A comparative study of extant and extinct toothed whales. Palaeogeography, Palaeoclimatology, Palaeoecology, 400:62–74.

Heyning, J.E. 1989. Comparative facial anatomy of beaked whales (Ziphiidae) and a systematic revision among the families of extant Odontoceti. Contributions in Science, Natural History Museum of Los Angeles County, 405:1–64.

Heyning, J.E. 1997. Sperm whale phylogeny revisited: analysis of the morphological evidence. Marine Mammal Science, 13(4):596–613.

Howell, A.B. 1927. Contribution to the anatomy of the Chinese finless porpoise *Neomeris phocaenoides*. Proceedings of The United States National Museum, 70(13):1–43.

Jefferson, T.A., and Newcomer, M.W. 1993. *Lissodelphis borealis*. Mammalian Species:1–6.

Kasuya, T. 1973. Systematic consideration of recent toothed whales based on the morphology of tympano-periotic bone. Scientific Reports of the Whales Research Institute, Tokyo, 25:1–103.

Kellogg, R. 1923. Description of an apparently new toothed cetacean from South Carolina. Smiths Misc Coll, 76:1–7.

Kellogg, R. 1936. A review of the Archaeoceti. Carnegie Institution of Washington publication, 482:1–366.

Klima, M., Oelschlaeger, H.A., and Wünsch, D. 1980. Morphology of the pectoral girdle in the Amazon dolphin Inia geoffrensis with special reference to the shoulder joint and the movements of the flippers. Zeitschrift Saugertierkunde, 45:288–309.

Kuzmin, A.A. 1976. Embryogenesis of the osseous skull of the sperm whale (Physeter macrocephalus Linnaeus, 1758). Investigations on Cetacea, 7:187–202.

Lambert, O. 2005. Phylogenetic affinities of the long-snouted dolphin *Eurhinodelphis* (Cetacea, Odontoceti) from the Miocene of Antwerp, Belgium. Palaeontology, 48(3):653–679.

Lambert, O. 2008. A new porpoise (Cetacea, Odontoceti, Phocoenidae) from the Pliocene of the North Sea. Journal of Vertebrate Paleontology, 28(3):863–872.

Lambert, O., G. Bianucci, M. Urbina, and J. H. Geisler. 2017. A new inioid (Cetacea, Odontoceti, Delphinida) from the Miocene of Peru and the origin of modern dolphin and porpoise families. Zoological Journal of the Linnean Society 179:919–946.

Leatherwood, S., Reeves, R.R., and Foster, L. 1983. Sierra Club handbook of whales and dolphins. Sierra Club Books, San Francisco.

Luo Z, Gingerich PD. 1999. Terrestrial Mesonychia to aquatic Cetacea: transformation of the basicranium and evolution of hearing in whales. University of Michigan Papers on Paleontology 31: 1–98.

Luo, Z., and Marsh, K. 1996. Petrosal (periotic) and inner ear of a Pliocene kogiine whale (Kogiinae, Odontoceti): implications on relationships and hearing evolution of toothed whales. Journal of Vertebrate Paleontology, 16(2):328–348.

Marsh, H., Lloze, R., Heinsohn, G.E., and Kasuya, T. 1989. Irrawaddy dolphin - *Orcaella brevirostris*, (Gray, 1866), p. 101–118. In Ridgway, S.H., and Harrison, S. (eds.), Handbook of marine mammals. Volume 4: river dolphins and the larger toothed whales.

McLeod SA, Whitmore FC, Jr., Barnes LG. 1993. Evolutionary relationships and classification. In: Burns JJ, Montague JJ and Cowles CJ, eds. The Bowhead whale: The Society for Marine Mammalogy. 45–70.

McGowen, M.R., Spaulding, M., Gatesy, J. 2009. Divergence date estimation and a comprehensive molecular tree of extant cetaceans. Molecular Phylogenetics and Evolution, 53:891–906.

McGowen, M.R., Montgomery, S.H., Clark, C, Gatesy, J. 2011. Phylogeny and adaptive evolution of the brain-development gene microcephalin (MCPH1) in cetaceans. BMC Evolutionary Biology, 11:98.

McGowen, M. R., Tsagkogeorga, G., Álvarez-Carretero, S., Dos Reis, M., Struebig, M., Deaville, R., Jepson, P.D., Jarman, S., Polanowski, A., Morin, P.A., Rossiter, S. J. 2020. Phylogenomic resolution of the cetacean tree of life using target sequence capture. Systematic Biology, 69(3):479–501.

Mead, J. 1989. Shepherd’s beaked whale *Tasmacetus shepherdi* Oliver, 1937. Handbook of marine mammals. Volume 4: river dolphins and the larger toothed whales., 4:309–320.

Mead, J.G. 1975. Anatomy of the external nasal passages and facial complex in the Delphinidae (Mammalia, Cetacea). Smithsonian Contributions to Zoology, 207:1–72.

Mead, J.G., and Fordyce, R.E. 2009. The therian skull: a lexicon with emphasis on the odontocetes. Smithsonian Contributions to Zoology, 627:1–248.

Messenger, S. 1994. Phylogenetic relationships of platanistoid river dolphins (Odontoceti, Cetacea): assessing the significance of fossil taxa. Contributions in Marine Mammal Paleontology Honoring Frank Whitmore Jr.Proceedings of the San Diego Society of Natural History:125–133.

Messenger, S.L., and McGuire, J.A. 1998. Morphology, molecules, and the phylogenetics of cetaceans. Systematic Biology, 47(1):90–124.

Miller, G.S. 1923. The telescoping of the cetacean skull. Smithsonian Miscellaneous Collections, 76(5):1–70.

Moore, J.C. 1968. Relationships among the living genera of beaked whales with classifications, diagnoses and keys. Fieldiana: Zo*ology,* 53(4):509–598.

Muizon C, de. 1984. Les vertébrés de la Formation Pisco (Pérou). Deuxième partie: Les Odontocètes (Cetacea, Mammalia) du Pliocène inférieur de Sud-Sacaco. Travaux de l’Institut Français d’Etudes Andines 27: 1–188.

Muizon, C. de. 1985. Nouvelles données sur le diphylétisme des Dauphins de rivière (Odontoceti, Cetacea, Mammalia). Comptes rendus l'Academie des Sciences series 2, 301:359–362.

Muizon C, de. 1987. The affinities of *Notocetus vanbenedeni*, an Early Miocene platanistoid (Cetacea Mammalia) from Patagonia, southern Argentina. American Museum Novitates 2904:1–27.

Muizon C, de. 1988. Les relations phylogénétiques des Delphinida. Annales de Paléontologie 74:159–227.

Muizon C, de. 1991. A new Ziphiidae (Cetacea) from the Early Miocene of Washington State (USA) and phylogenetic analysis of the major groups of odontocetes. Bulletin du Muséum national d’Histoire naturelle, Paris 12:279–326.

Muizon, C. de. 1994. Are the squalodonts related to the platanistoids? Proceedings of the San Diego Society of Natural History, 29:135–146.

Murakami, M., Shimada, C., Hikida, Y., and Hirano, H. 2012a. A new basal porpoise, *Pterophocaena nishinoi* (Cetacea, Odontoceti, Delphinoidea), from the upper Miocene of Japan and its phylogenetic relationships. Journal of Vertebrate Paleontology, 32(5):1157–1171.

Murakami, M., Shimada, C., Hikida, Y., and Hirano, H. 2012b. Two new extinct basal phocoenids (Cetacea, Odontoceti, Delphinoidea), from the upper Miocene Koetoi Formation of Japan and their phylogenetic significance. Journal of Vertebrate Paleontology, 32(5):1172–1185.

Murakami, M., Shimada, C., Hikida, Y., Soeda, Y., and Hirano, H. 2014. *Eodelphis kabatensis*, a new name for the oldest true dolphin *Stenella kabatensis* Horikawa, 1977 (Cetacea, Odontoceti, Delphinidae), from the upper Miocene of Japan, and the phylogeny and paleobiogeography of Delphinoidea. Journal of Vertebrate Paleontology, 34(3):491–511.

Noble, B., and Fraser, F. 1971. Description of a skeleton and supplementary notes on the skull of a rare porpoise *Phocoena sinus* Norris & McFarland 1958. Journal of Natural History, 5(4):447–464.

Norris, K.S. 1964. Some problems of echolocation in cetaceans, p. 317–336. In Tavolga, W.N. (ed.), Marine bio-acoustics. MacMillan, New York.

Norris, K.S., and Harvey, G.W. 1972. A theory for the function of the spermaceti organ of the sperm whale (Physeter catodon L). NASA Special Publication, 262:397.

Reeves, R., Stewart, B., Clapham, P., and Powell, J. 2002. Marine mammals of the world. Chanticleer Press, Inc, New York.

Rice, D.W., and Wolman, A.A. 1990. The stomach of *Kogia breviceps*. Journal of Mammalogy:237–242.

Rommel, S. 1990. Osteology of the bottlenose dolphin, p. 29–49. In Leatherwood, S., and Reeves, R.R. (eds.), The bottlenose dolphin. Academic Press Inc, San Diego, New York.

Sanders, A.E., and Barnes, L.G. 2002. Paleontology of the late Oligocene Ashley and Chandler Bridge formations of South Carolina, 2: Micromysticetus rothauseni, a primitive cetotheriid mysticete (Mammalia: Cetacea). Smithsonian Contributions to Paleobiology(93):271–293.

Schenkkan, E. 1971. The occurrence and position of the “connecting sac” in the nasal tract complex of small odontocetes (Mammalia, Cetacea). Beaufortia, 19(246):37–43.

Schulte, H.v.W. 1917. The skull of Kogia breviceps Blainv. Bulletin of the American Museum of Natural History(37):361–404.

Tanaka Y., Abella J., Aguirre-Fernández, G., Gregori M., Fordyce R.E. 2017. A new tropical Oligocene dolphin from Montañita/Olón, Santa Elena, Ecuador. PLoS ONE 12(12): e0188380.

Tanaka, Y., and Fordyce, R.E. 2014. Fossil dolphin *Otekaikea marplesi* (latest Oligocene, New Zealand) expands the morphological and taxonomic diversity of Oligocene cetaceans. PLoS ONE, 9(9):e107972.

Tanaka, Y., and Fordyce, R.E. 2015. A new Oligo-Miocene dolphin from New Zealand: *Otekaikea huata* expands diversity of the early Platanistoidea. Palaeontologia Electronica, 18(2.23A):1–71.

True, F.W. 1904. The whalebone whales of the western North Atlantic compared with those occurring in European waters with some observations on the species of the North Pacific. Smithson Contribution to Knowledge, 33:1–332.

Van Valen, L. 1968. Monophyly or diphyly in the origin of whales. Evolution:37–41.

Whitmore, F.C., and Sanders, A.E. 1977. Review of the Oligocene Cetacea. Systematic Zoology, 25(4):304–320.

Yablokov, A. 1964. Convergence or parallelism in the evolution of cetaceans. International Geology Review, 7(8):1461–1468.

Yamada, M. 1953. Contribution to the anatomy of the organ of hearing of whales. Scientific Reports of the Whales Research Institute, 8:1–79.

Zhou, K. 1982. Classification and phylogeny of the superfamily Platanistoidea, with notes on evidence of the monophyly of the Cetacea. Scientific Reports of the Whales Research Institute Tokyo, 34:93–108.
